# Ligand-based design, synthesis, computational insights, and *in vitro* studies of novel *N*-(5-Nitrothiazol-2-yl)-carboxamido derivatives as potent inhibitors of SARS-CoV-2 main protease

**DOI:** 10.1080/14756366.2022.2105322

**Published:** 2022-07-31

**Authors:** Mohamed Elagawany, Ayman Abo Elmaaty, Ahmed Mostafa, Noura M. Abo Shama, Eman Y. Santali, Bahaa Elgendy, Ahmed A. Al-Karmalawy

**Affiliations:** a Department of Pharmaceutical Chemistry, Faculty of Pharmacy, Damanhour University, Damanhour, Egypt; b Department of Medicinal Chemistry, Faculty of Pharmacy, Port Said University, Port Said, Egypt; c Center of Scientific Excellence for Influenza Viruses, National Research Centre, Cairo, Egypt; d Institute of Medical Microbiology, German Center for Infection Research (DZIF), Justus-Liebig University Giessen, Giessen, Germany; e Department of Pharmaceutical Chemistry, College of Pharmacy, Taif University, Taif, Saudi Arabia; f Center for Clinical Pharmacology, Washington University School of Medicine, University of Health Sciences, St. Louis, MO, USA; g Chemistry Department, Faculty of Science, Benha University, Benha, Egypt; h Department of Pharmaceutical Medicinal Chemistry, Faculty of Pharmacy, Horus University-Egypt, New Damietta, Egypt

**Keywords:** *N*-(5-nitrothiazol-2-yl)-carboxamido derivatives, *in vitro*, anti-SARS-CoV-2 Mpro, *in silico*, SAR

## Abstract

The global outbreak of the COVID-19 pandemic provokes scientists to make a prompt development of new effective therapeutic interventions for the battle against SARS-CoV-2. A new series of *N*-(5-nitrothiazol-2-yl)-carboxamido derivatives were designed and synthesised based on the structural optimisation principle of the SARS-CoV Mpro co-crystallized WR1 inhibitor. Notably, compound **3b** achieved the most promising anti-SARS-CoV-2 activity with an IC_50_ value of 174.7 µg/mL. On the other hand, compounds **3a**, **3b**, and **3c** showed very promising SARS-CoV-2 Mpro inhibitory effects with IC_50_ values of 4.67, 5.12, and 11.90 µg/mL, respectively. Compound **3b** docking score was very promising (−6.94 kcal/mol) and its binding mode was nearly similar to that of WR1. Besides, the molecular dynamics (MD) simulations of compound **3b** showed its great stability inside the binding pocket until around 40 ns. Finally, a very promising SAR was concluded to help to design more powerful SARS-CoV-2 Mpro inhibitors shortly.

## Graphical Abstract

**Figure:**
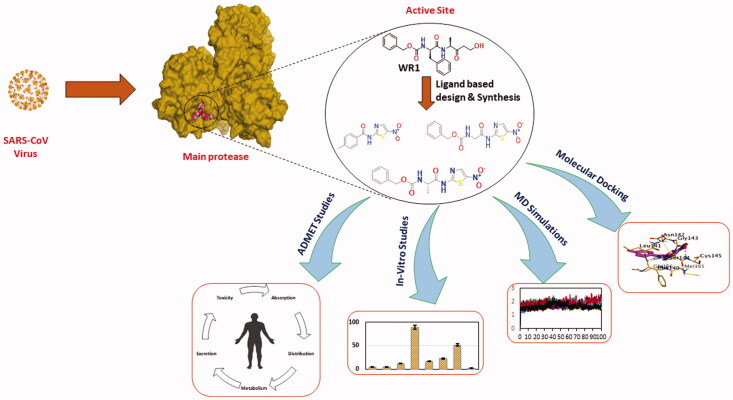

**HIGHLIGHTS**
- A new series of *N*-(5-nitrothiazol-2-yl)-carboxamido derivatives were designed and synthesised based on the structural optimisation principle.
- *In vitro* antiviral activities against SARS-CoV-2 using SARS-CoV-2 cell-based inhibitory assay.
- The anticipated inhibitory effects of the synthesised compounds (**3a–g**) towards the SARS-CoV-2 Mpro enzyme were emphasised by using the SARS-CoV-2 Mpro assay.
- Molecular docking studies, molecular dynamics simulations for 100 ns, and MM-GBSA calculations were carried out for the newly synthesised compounds (**3a–g**) compared to the co-crystallized inhibitor (WR1).
- ADMET and toxicity *in silico* studies were applied for the designed derivatives.
- Finally, our interesting work rationale helped to conclude a very promising structure-activity relationship (SAR) finding.

## 1. Introduction

The COVID-19 global outbreak is attributed to SARS-CoV-2^1^. Owing to its overwhelming expansion and spreading, the virus caused an unprecedented global health crisis. Subsequently, the World Health Organisation (WHO) officially claimed that COVID-19 is pandemic in March 2020. SARS-CoV-2 has reached over 170 countries and has adversely impacted over 235 million individuals with a death toll nearing 5.2 million as of 22 November 2021^1^^,^^2^. Besides, the incubation period of SARS-CoV-2 is nearly 2–14 days and can be extended up to 24 days. The virus's long incubation period, and its possible asymptomatic nature, could be in charge of infections spreading. The fast rise in COVID-19 cases increases the need for effective interventions^3–6^.

Furthermore, the virus belongs to the Coronaviridae family and generally coronaviruses can be classified to four genera, gamma-coronavirus (*γ*-CoV), delta-coronavirus (*δ*-CoV), alpha-coronavirus (*α*-CoV), and beta-coronavirus (*β*-CoV). Both *α*- and *β*-species mainly hit mammals, while *γ*- and *δ*- species hit birds^7^. Notably, it was confirmed that SARS‐CoV‐2 shares almost 80% of the genome with SARS‐CoV^8^. Infection by SARS-CoV-2 is transmitted mainly through human-to-human contact from respiratory droplets. The viral infection varies in severity from asymptomatic to threatening fatal disease. Consequently, the most common symptoms include headache, fever, non-productive cough, fatigue, and dyspnoea. Patients with severe disease may develop viral pneumonia, hypoxia, and acute respiratory distress. So, intubation and mechanical ventilation are required^1^. Additionally, neurological symptoms including skeletal muscle injury, acute cerebrovascular diseases, consciousness impairment, and loss of smell and/or taste could be manifested by SARS-CoV-2 infection^9^^,^^10^.

Additionally, coronaviruses belong to RNA viruses [single-stranded positive-sense (+)] that are distinctly prevalent in wildlife and humans. Notably, coronaviruses have the most enormous known RNA genomes. Hence, the virus’s two encoded overlapping open-reading frames are translated into the two polyproteins named; pp1a and pp1ab. So, these polyproteins are processed further to give rise to four structural proteins and sixteen non-structural proteins (nsps)^11^. Subsequently, the virus replicase polyprotein is processed by two distinct cysteine proteases; the papain-like protease (PLpro) and the main protease (Mpro)^12^^,^^13^. The proteolytic refining of the sixteen nsps by PLpro and 3CLpro is crucial for virus maturation and replication, and therefore PLpro and 3CLpro emerged as key druggable targets^14–18^.

For the sake of achieving rapid therapeutic interventions, a handful set of repurposed drugs^19^^,^^20^ like chloroquine, hydroxychloroquine, and remdesivir, has been used frequently for COVID-19 treatment^3^^,^^4^. Although remdesivir, which gained urgent approval, hydroxychloroquine and nafamostat are viewed as outstanding therapeutic candidates, their low clinical effects and adverse side effects warrant the search for more effective and safer treatments^21^. Several SARS-CoV-2 druggable targets were elucidated such as Mpro, spike (S) protein, papain-like protease (PLpro), and RNA-dependent RNA polymerase. The viral Mpro is regarded as an outstanding target for druggability^3^^,^^22^.

**Figure 1.**
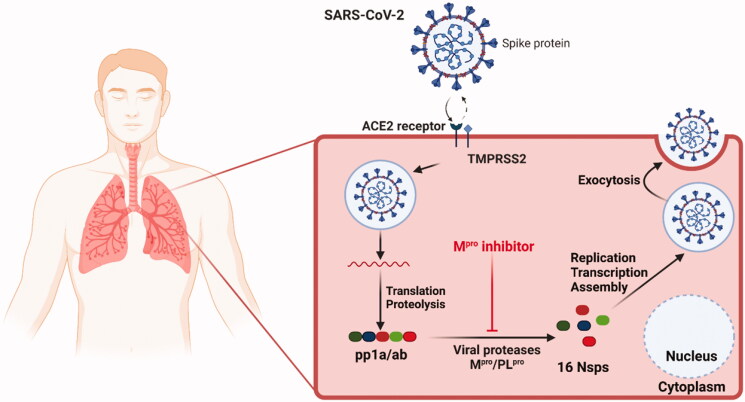
Schematic diagram showing SARS-CoV-2 host, its transmission, and the virus Mpro as a promising druggable target of interest in an infected cell.

The Mpro enzyme is one of the best coronavirus drug targets due to the resemblance in their active site and mechanisms with β-Coronaviruses from previous epidemics; SARS-CoV and MERS-CoV (Middle East respiratory syndrome coronavirus)^23^. Mpro is a preserved drug target without a human homolog, hence lowering the possibility of accidentally targeting host proteins. Therefore, Mpro is perceived as a potential target for broad-spectrum drug development^23^^,^^24^. It is worth mentioning that findings propose that SARS-CoV-2 possesses the power to utilise human angiotensin-converting enzyme 2 (ACE2) receptors in the seek of cell entrance as displayed in Figure 1^7^^,^^16^.

Despite all efforts and attempts to find a treatment for SARS-CoV-2 infection, rising issues of COVID-19 mortality and morbidity are still encountered globally. Although vaccines have been developed, efficient and safe drugs are urgently needed^5^^,^^10^^,^^25^.

Lately, a new synthetic nucleoside derivative prodrug, named molnupiravir, was approved in the U.K for COVID-19 treatment. Molnupiravir acts by copying errors during RNA virus replication^26^. It is an active orally RdRp inhibitor with reasonable pharmacokinetic features. It has gained significant attention for its capability to inhibit the spreading of SARS-CoV-2, with a remarkable reduction in the viral load and quick recovery time^27^. A single-dose administration of molnupiravir produces a mean *C*_max_ of 13.2 ng/mL and *t*_max_ between 0.25 and 0.75 h with a biological t_1/2_ of 7 h. It was suggested that molnupiravir has no accumulative toxicity and that was assured by its area under the plasma concentration versus time following multiple doses, increases with no accumulation in a dose-proportional manner^27^. Moreover, molnupiravir could exhibit rapid onset, a wide therapeutic window, and fewer side effects with good tolerability and safety profile. Hence, it can be considered a very promising therapeutic intervention against SARS-CoV-2^27^. Additionally, the oral antiviral drug, named PF-07321332, was developed by Pfizer For COVID-19 treatment as well. PF-07321332 acts as an active Mpro inhibitor of the virus^26^. Protease inhibitors act by interrupting the protease enzyme cutting, thus, the polypeptide processing to smaller protein is blocked. PF-07321332 is co-administered with ritonavir in low doses as a booster to enhance the PF-07321332 bloodstream levels^28^. The combination of ritonavir/PF-07321332 was marketed as paxlovid^26^.

**Scheme 1.**
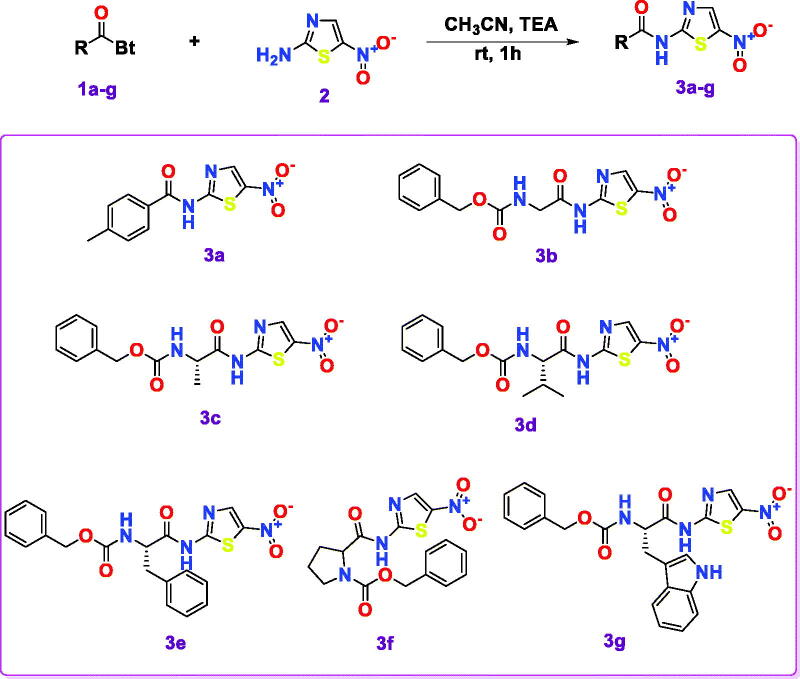
Chemical synthesis of the designed target compounds (**3a–g**) attempted to combat COVID-19.

In recent months, many researchers disclosed the discovery of potent inhibitors for SARS-CoV-2 using molecular docking and dynamics *in silico* approaches^19^^,^^29–36^. Moreover, the literature revealed that some novel chemically synthesised compounds were designed and evaluated biologically as outstanding inhibitors of SARS-CoV-2 Mpro^37–40^. Obviously, the *N*-heterocyclic scaffolds commonly exhibit a pivotal function and exert an advanced biological activity against SARS-CoV-2. Hence, a promising therapeutic intervention for COVID-19 treatment can be acquired^25^. Therefore, in the current work, we aimed to synthesise a series of *N*-heterocyclic scaffold derivatives that have the same pharmacophoric features of SARS-CoV Mpro native inhibitor (N3) as depicted in Scheme 1. Thus, the virus Mpro was targeted revealing the potential of the synthesised compounds as promising candidates for COVID-19 treatment using both *in vitro* and *in-silico* approaches for their assessment.

### 1.1. The rationale for work design

**Figure 2.**
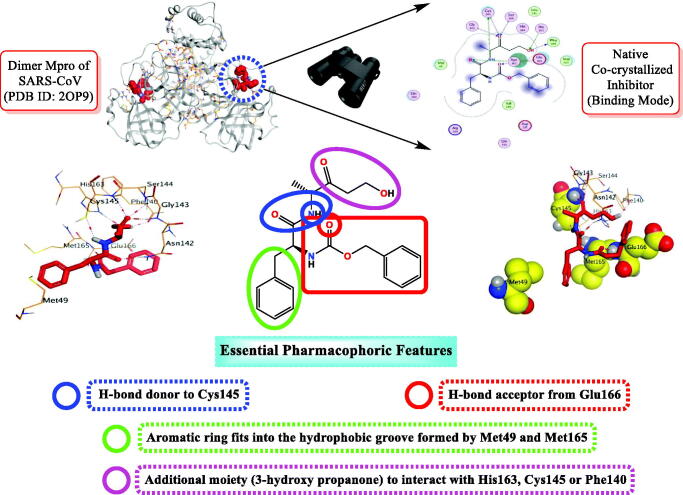
The rationale work design shows the identification of the essential pharmacophores acquired by the SARS-CoV Mpro co-crystallized inhibitor.

WR1 is the three-letter code of the native inhibitor of SARS-CoV Mpro downloaded from PDB with ID 2OP9^41^. Observing the native inhibitor (WR1) binding mode at SARS-CoV Mpro, we can conclude that it could be stabilised within its binding pocket via the following essential pharmacophoric features (Figure 2);

- H-bond donor (NH) to compose an H-bond with Cys145 amino acid.
- H-bond acceptor (CO) to compose an H-bond with Glu166 amino acid.
- Aromatic moiety to occupy the hydrophobic groove composed of the amino acids; Met49 and Met165.
- Additional moiety (3-hydroxy propanone) to interact with His163, Cys145, or Phe140 amino acids.

**Figure 3.**
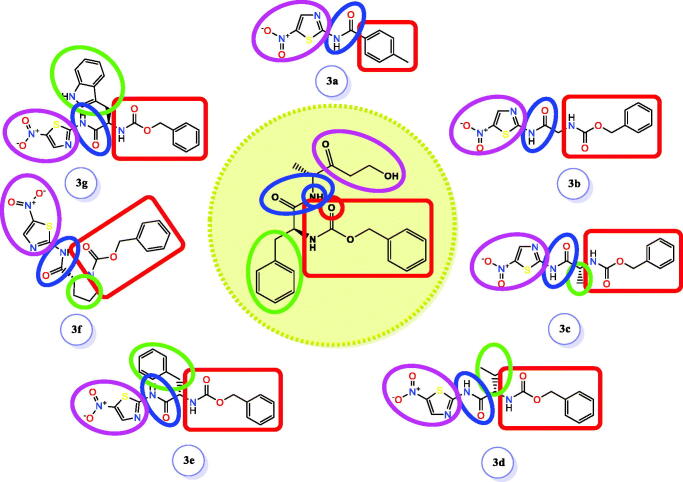
Schematic representation describing the achievement of the previously identified pharmacophoric features of SARS-CoV Mpro inhibitor in the newly designed drug candidates (**3a–g**).

**Figure 4.**
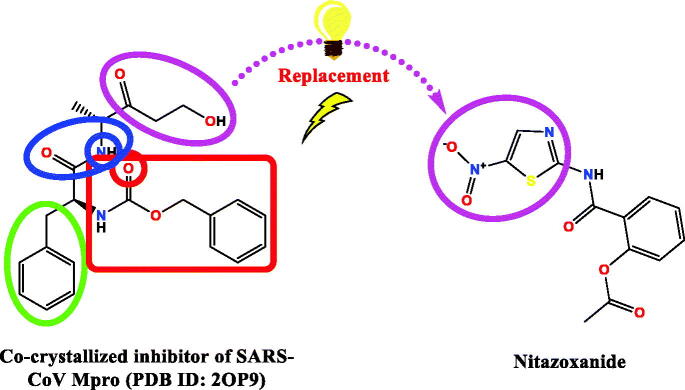
The replacement of the 3-hydroxy propanone moiety of the co-crystallized SARS-CoV Mpro inhibitor that interacts with His163, Cys145, or Phe140 with 5-nitrothiazole moiety which was extracted from the potent anti-SARS-CoV-2, nitazoxanide.

On the other hand, it is worth mentioning that the inhibitor-binding site is located at Cys–His dyad which composes the catalytic cleft located between the SARS-CoV-2 Mpro domains I and II. Herein, the authors analysed the pharmacophoric features of SARS-CoV Mpro co-crystallized inhibitor (WR1) to synthesise a new series of compounds (**3a–g**) using the ligand-based design approach^42^ and based on the structural optimisation principle. In addition, taking into account the close structural similarity within the two strains of SARS-CoV (1 and 2)^4^^,^^10^, we dedicated our efforts to synthesising a novel series of *N*-(5-nitrothiazol-2-yl)-carboxamido derivatives as significant inhibitors of SARS-CoV-2 Mpro (Figure 3), where;

- We kept the H-bond donor moiety (amidic group) such as the co-crystallized inhibitor (WR1).
- We kept the H-bond acceptor moiety (benzyl carbamate) such as the co-crystallized inhibitor (WR1) except for compound **3a**.
- We modified the aromatic ring that fits within the hydrophobic groove composed of the amino acids; Met49 and Met165 to other different moieties with different sizes (methyl, isopropyl, benzyl, pyrrole, or indole substituents) in compounds **3c**, **3d**, **3e**, **3f**, and **3g**, respectively. Also, we removed this moiety in both compounds **3a** and **3b**.
- We replaced the previously mentioned additional moiety (3-hydroxy propanone) that interacts with His163, Cys145, or Phe140 with 5-nitrothiazole moiety which was extracted from nitazoxanide (Figure 4) which was later approved to possess potent antiviral activities against hepatitis B and C, influenza A, and coronaviruses. Recently, nitazoxanide was evaluated against SARS-CoV-2 through *in vitro* assessment which confirmed its promising activity (EC_50_ of 2.12 μM)^43^.

Based on the aforementioned rationale, we were able to assess the impact of the discussed modifications on the potential of the synthesised candidates to get a lead compound and obtain a reasonable structure-activity relationship (SAR) which could aid medicinal chemists to design more promising anti-SARS-CoV-2 drug candidates soon as well.

## 2. Results and discussion

### 2.1. Chemistry

Compounds **3a–g** were synthesised by treating *N*-acyl benzotriazoles (**1a–g**) with 5-nitrothiazol-2-amine (**2**) at room temperature in the presence of triethylamine in acetonitrile for 1 h following the reported methodologies^44^^,^^45^.

### 2.2. *In vitro* studies

#### 2.2.1. SARS-CoV-2 inhibitory assay

**Figure 5.**
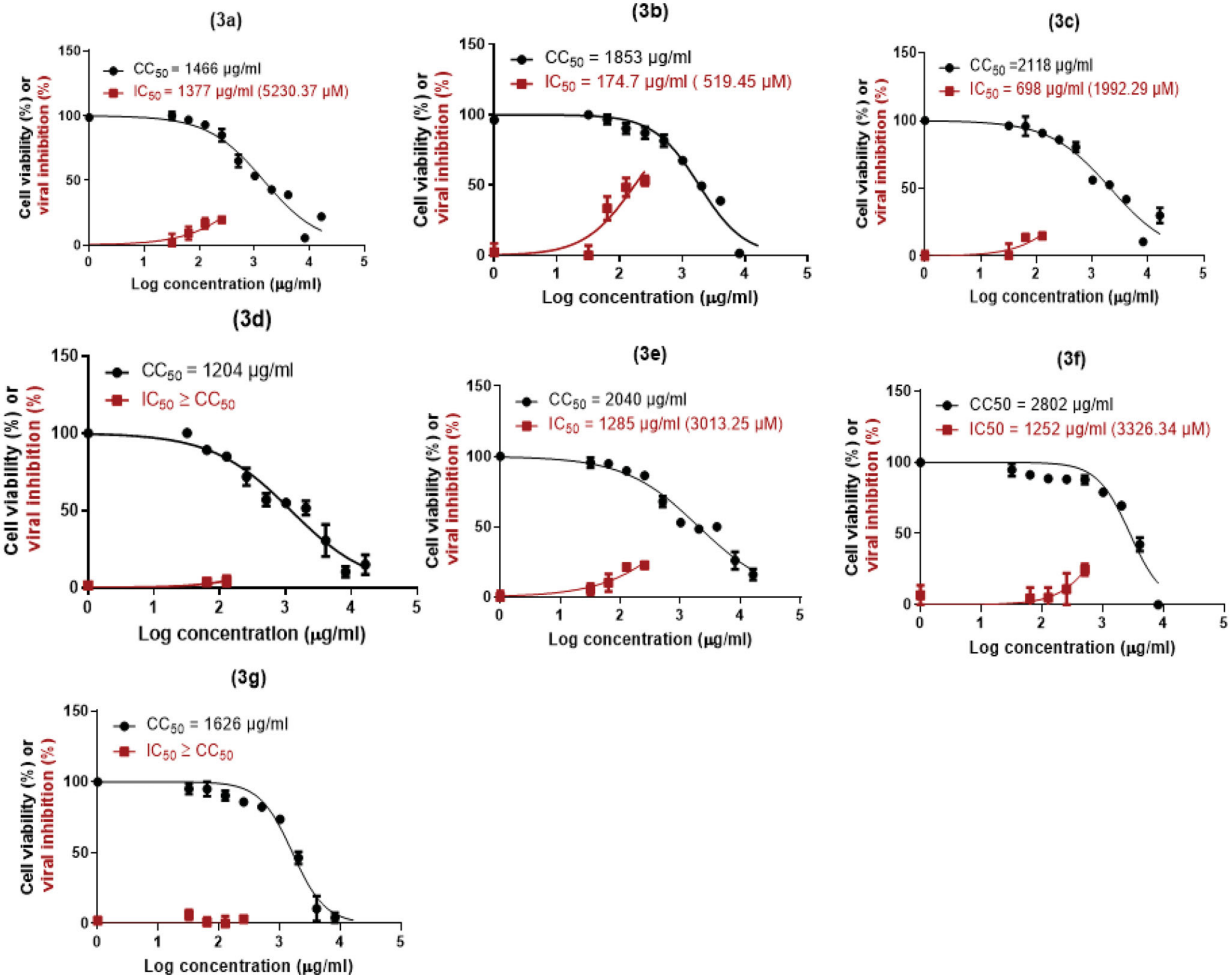
Cytotoxicity concentration 50 (CC_50_) of the newly designed and synthesised SARS-CoV Mpro analogs (**3a–g**) on Vero E6 cells. Besides, inhibitory concentration (IC_50_) to estimate the antiviral activity against SARS-CoV-2 [*h*CoV-19/Egypt/NRC-03/2020 (Accession Number on GSAID: EPI_ISL_430820)] using Vero E6 cells.

To investigate the anti-SARS-CoV-2 activity in Vero E6 cells, the cytotoxicity of the tested compounds was assessed in Vero E6 cells via MTT test and the results unravelled that the cytotoxic concentration 50 (CC_50_) values were 1466 µg/mL (**3a**), 1853 µg/mL (**3b**), 2118 µg/mL (**3c**), 1204 µg/mL (**3d**), 2040 µg/mL (**3e**), 2802 µg/mL (**3f**), and 1626 µg/mL (**3g**) (Figure 5). Furthermore, the antiviral activities were estimated using the dose-response curves. The result showed that the concentrations that induce inhibition to 50% of the investigated cells (IC_50_) by the tested compounds were 1377 µg/mL (**3a**), 174.7 µg/mL (**3b**), 698 µg/mL (**3c**), 1285 µg/mL (**3e**), and 1252 µg/mL (**3f**) (Figure 5). For all tested compounds, the IC_50_ values were estimated by plotting log inhibitory concentrations (X-axis) against normalised response (Y-axis), (variable slope) utilising GraphPad Prism software (version 5.01) nonlinear regression analysis. However, compounds (**3d**) and (**3g**) displayed IC_50_ values higher than their corresponding CC_50_ values neglecting their applicability as anti-SARS-CoV-2. Consequently, compound **3b** showed the best selectivity index (SI = CC_50_/IC_50_) with a SI = 10, followed by **3c** with a SI value of 3.

#### 2.2.2. SARS-CoV-2 Mpro inhibitory assay (cell-based)

**Figure 6.**
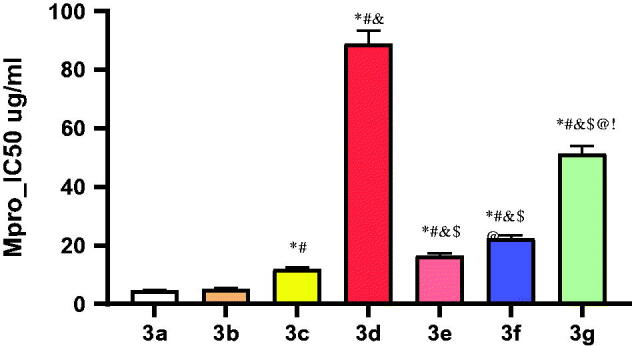
Mpro inhibitory concentration 50 (IC_50_) against SARS-CoV-2 for the synthesised compounds (**3a–g**), where compounds **3a**, **3b**, and **3c** unravelled so outstanding SARS-CoV-2 Mpro inhibitory effects with IC_50_ 4.67, 5.12, and 11.90 µM, respectively. *p ˂ 0.05 compared to **3a**. ^#^*p* ˂ 0.05 compared to **3b**. ^&^*p* ˂ 0.05 compared to **3c**. ^$^*p* ˂ 0.05 compared to **3d**. ^@^*P* ˂ 0.05 compared to **3e**. ^!^*p* ˂ 0.05 compared to **3g**.

The anticipated inhibitory effects of the synthesised derivatives (**3a-g**) towards the SARS-CoV-2 Mpro enzyme were emphasised by using the SARS-CoV-2 Mpro assay. Out of the synthesised compounds, compounds **3a**, **3b**, and **3c** unravelled so outstanding SARS-CoV-2 Mpro inhibitory effects with IC_50_ values of 4.67, 5.12, and 11.90 µg/mL, respectively, as displayed in Figure 6 as well as the Supplementary Data (Supplementary Table 1). It is worth mentioning that among these promising compounds, compounds **3a** and **3b** fulfilled the best inhibitory activity against SARS-CoV-2 Mpro with very promising IC_50_ values.

### 2.3. In silico studies

#### 2.3.1. Molecular docking studies

**Figure 7.**
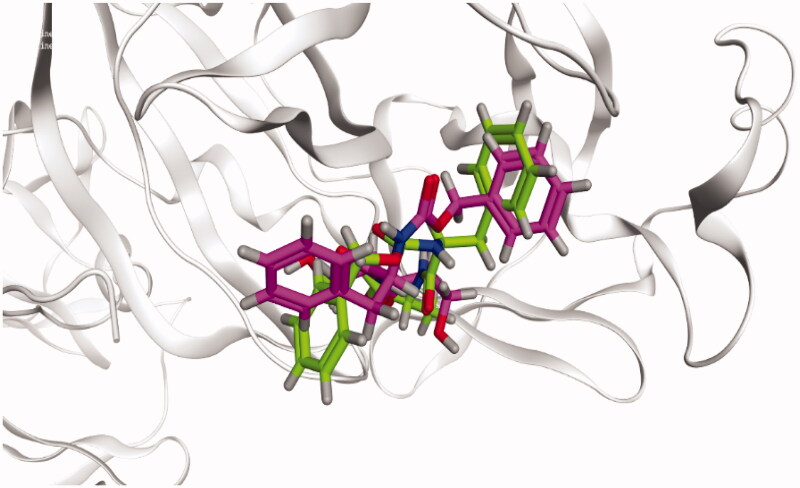
3D diagram unveiling the native ligand WR1 (Green), and redocked WR1 (Violet) superimposition at SARS-CoV Mpro with PDB: 2OP9 for MOE program validation.

At the beginning of the docking process, to assure the accuracy of the docking protocol, the MOE program was validated. So, the program validation was initiated by the native ligand (WR1) re-docking against the SARS-CoV Mpro target receptor^46–48^. A valid docking protocol was ensured by getting a low RMSD value (1.33 Å) between the re-docked conformer and the co-crystallized conformer of WR1 as shown in Figure 7 ^49^^,^^50^.

**Figure 8.**
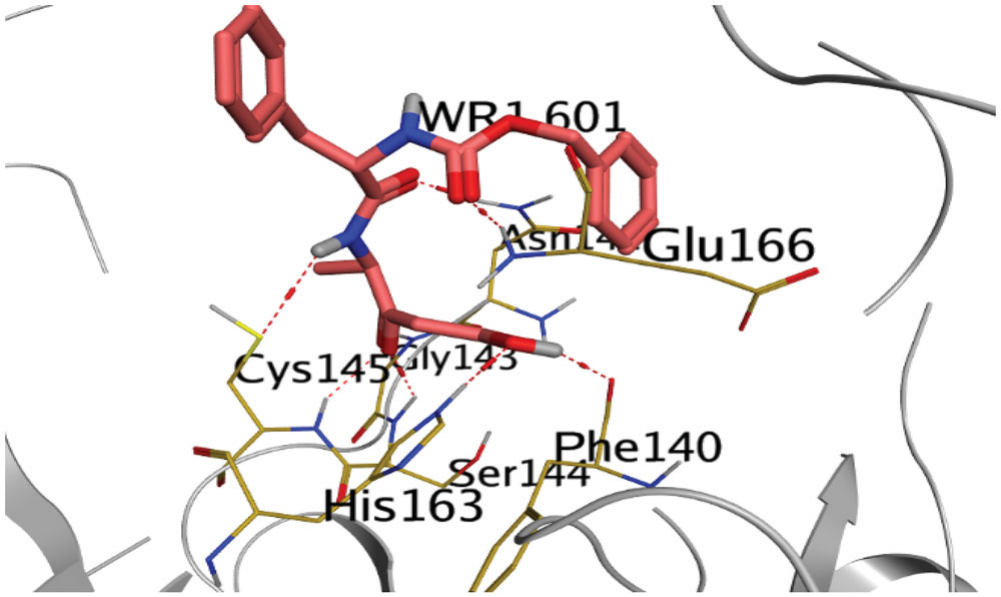
Native co-crystallized WR1 inside SARS-CoV Mpro active site with PDB: 2OP9. The red dashed lines stand for hydrogen bonds.

WR1, as a native co-crystallized ligand, formed hydrogen bonds with Phe140 and His163 through the hydroxyl group of the oxopentan-2-yl moiety of WR1 at distances 2.79 and 2.91 Å, respectively. However, the docked WR1 formed hydrogen bonds with Ser144, Cys145, and Gly143 through the carbonyl group of the oxopentan-2-yl moiety of WR1 at distances 2.91, 2.94, and 2.87 Å, respectively. Moreover, the carbamate moiety of WR1 interacted with Glu166 via a hydrogen bond at a distance of 2.92 Å, but the amide moiety of WR1 formed a hydrogen bond with Asn142 and Cys145 at distances 2.95 and 3.49 Å, respectively (Figure 8).

**Figure 9.**
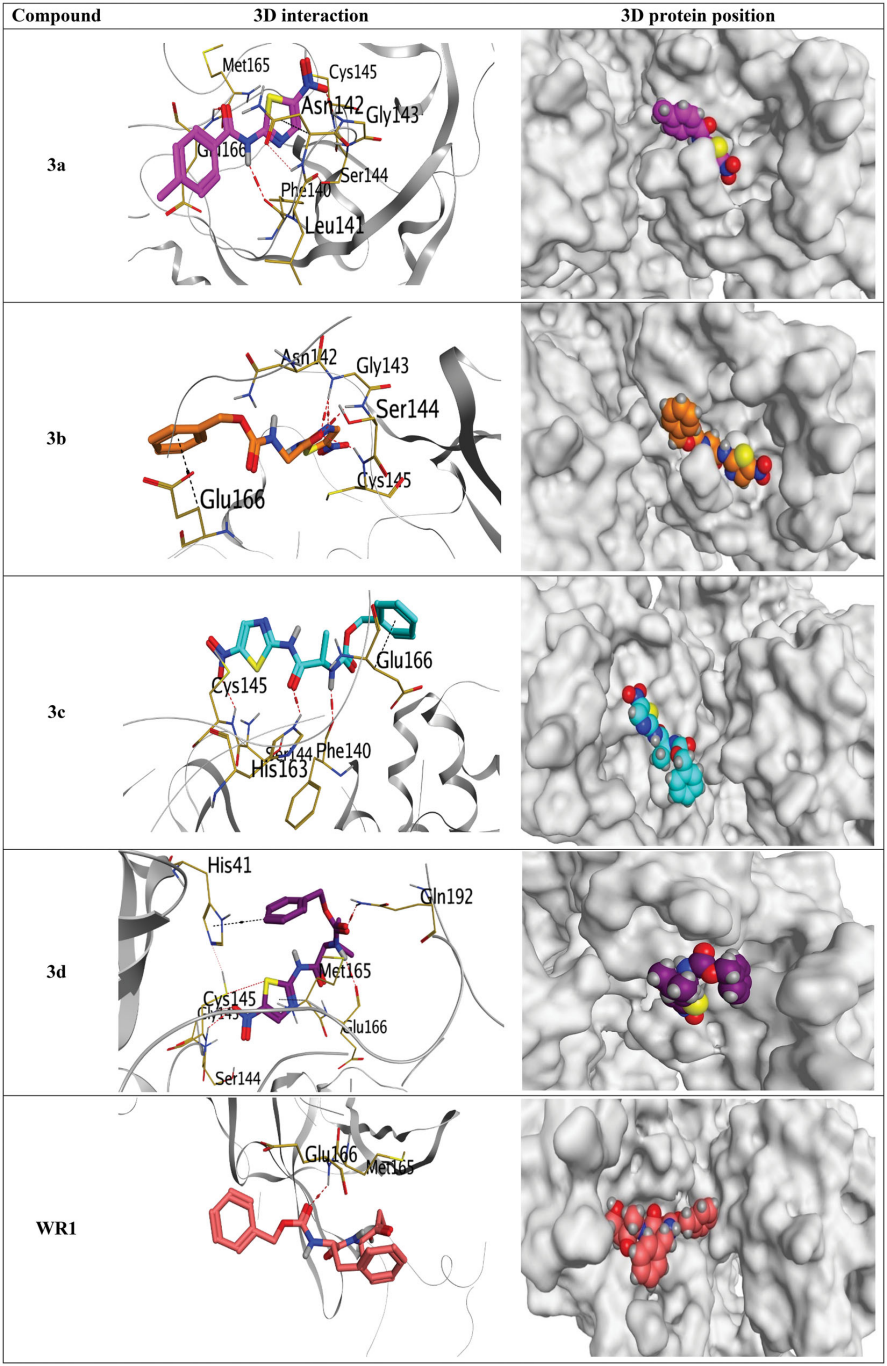
3D pictures of the synthesised compounds representing the binding interactions and positioning at the SARS-CoV Mpro pocket, with the co-crystallized redocked ligand (WR1). H-bonds were described by red dashed lines while H-pi bonds by black ones.

So, by analysing the docking depicted in Table 1 and Figure 9 of our synthesised compounds (**3a–g**) against Mpro pockets of SARS-CoV, taking into consideration the pharmacophoric features discussed before, we can conclude the following:

**Table 1.** Binding interaction scores, RMSD, amino acids, and bond types of the synthesised compounds (**3a–g**) inside the SARS-CoV-2 Mpro pocket of the co-crystallized WR1 inhibitor.

Compound	Score^a^	RMSD	Interactions	Bond type	Distance Å
**3a**	−5.43	1.45	PHE140 GLU166 GLY143 CYS145 ASN142	H Donor H Acceptor H Acceptor H Acceptor pi-H	3.18 3.12 2.93 3.05 3.80
**3b**	−6.94	1.41	GLY143 GLY143 SER144 CYS145 GLU166	H Acceptor H Acceptor H Acceptor H Acceptor pi-H	3.18 3.08 3.03 3.03 3.63
**3c**	−6.07	1.17	PHE140 CYS145 HIS163 GLU166	H Donor H Acceptor H Acceptor pi-H	3.00 3.39 3.14 3.98
**3d**	−7.01	3.01	CYS145 GLU166 GLY143 CYS145 GLN192 HIS41 MET165	H Donor H Donor H Acceptor H Acceptor H Acceptor H-pi pi-H	3.99 2.97 3.16 3.28 2.87 3.81 4.10
**3e**	−6.44	2.09	GLU166 GLY143 CYS145	H Donor H Acceptor H Acceptor	3.34 2.94 2.88
**3f**	−6.59	2.83	PHE140 CYS145 PHE140 GLU166 MET49	H Donor H Donor H Donor H Acceptor pi-H	3.25 3.65 3.21 3.38 4.06
**3g**	−6.80	1.71	GLU166 GLY143 SER144 CYS145	H Donor H Acceptor H Acceptor H Acceptor	3.51 3.22 3.28 3.10
WR1	−6.52	1.53	GLU166	H Acceptor	2.98

^a^S: The compound score inside the binding pocket (kcal/mol).

The redocked co-crystallized ligand, WR1, unveiled binding energy of −6.52 kcal/mol. It forms only one hydrogen bond with Glu166 through its carbamate moiety at a distance of 2.98 Å. However, taking them as representative examples with high anticipated intrinsic activity, compound **3a** has a binding interaction score of −5.43 kcal/mol towards Mpro pockets of SARS-CoV. The amide nitrogen of compound **3a** forms a hydrogen bond with Phe140 at a distance of 3.18 Å, whereas the nitro group forms a hydrogen bond with Cys145 at a distance of 3.05 Å. Moreover, the oxygen of the amide group of compound **3a** interacts with Glu166 through a hydrogen bond at a distance of 3.12 Å. Moreover, compound **3b** has a binding interaction score of −6.94 kcal/mol towards Mpro pockets of SARS-CoV. The phenyl ring of compound **3 b** forms a pi-H bond with Glu 166 at a distance of 3.63 Å. Whereas, the oxygen of the amide group of compound **3b** binds with Cys145 via hydrogen bond at a distance of 3.03 Å. Furthermore, compound **3c** has a binding interaction score of −6.07 kcal/mol towards Mpro pockets of SARS-CoV-2. The nitro group and the oxygen of amide moiety at the thiazole ring of compound **3c** were capable of composing H-bond with the amino acids; Cys145 and His163 at 3.39 and 3.14 Å, respectively, the two main amino acids composing the SARS-CoV-2 Mpro catalytic dyad^51^ indicating anticipated significant intrinsic activity against SARS-CoV-2. Besides, the carbamate nitrogen of compound **3c** forms H-bond with Phe144 with a distance of 3 Å, whereas the phenyl ring of Compound **3c** forms a pi-H bond with Glu166 with a distance of 3.98 Å. Moreover, the 2 D interactions of the newly designed hits (**3a–g**) were described in the Supplementary Data (Supplementary Table 2).

#### 2.3.2. Molecular dynamics (MD) simulations

To record the behaviour of the examined candidates inside the binding pocket of SARS-CoV during a time of 100 ns and using the same criteria for the physiological environment, MD simulations were performed accordingly. All of the seven docked complexes along with the co-crystallized WR1 inhibitor—as a standard—were subjected to MD simulations for 100 ns.

##### 2.3.2.1. RMSD and RMSF analysis

To compare the degree of deviation for the complexed protein structure related to its initial native form quantitatively, the RMSD was studied. This helps to investigate the system’s overall stability through the simulation time.

**Figure 10.**
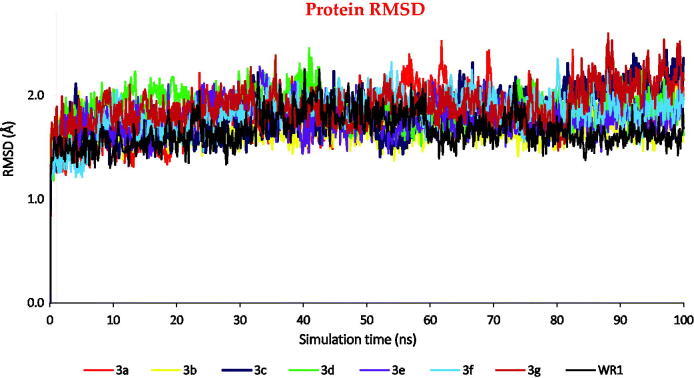
The RMSD of the C_α_ atoms of the complexes (**3a**–**g** and WR1) for the SARS-CoV protein against the time of simulation (100 ns).

The RMSD of the eight complexes showed good stability behaviours all over the simulation time with RMSD values in the range of (0.7–1.3) Å (Figure 10).

The Root Mean Square Fluctuation (RMSF) is useful to show the local changes that occur in the protein structure. In addition, it clarifies the degree of the protein residues’ flexibility through the simulation. The RMSF of the eight complexes was reported in the Supplementary Data (Supplementary Figure 1). The residue from 0 to 301 represents chain A, and residues from 302 to 602 represent chain B of the dimer. The most fluctuation was within the 0–3 Å range, the only exception is for terminal Ala0, Ser1, and Ser301 from both chains were found to fluctuate at around 3.10–3.30 Å.

Additionally, snapshots at 0, 50, and 100 ns for 3a-2OP9, 3b-2OP9, 3c-2OP9, and WR1-2OP9 complexes were represented in the Supplementary Data (Supplementary Figure 2).

**Figure 11.**
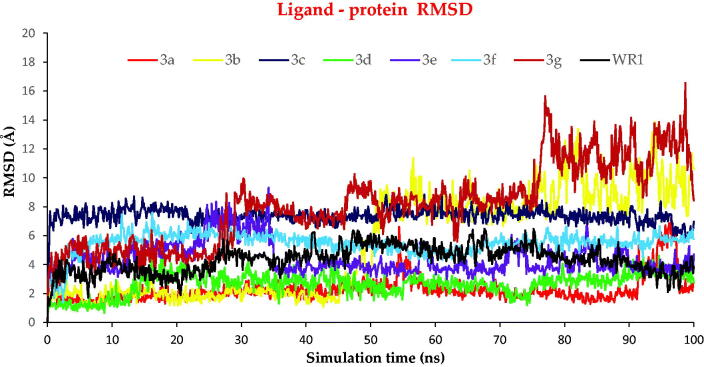
The RMSD of ligands (**3a**–**g** and WR1) for the SARS-CoV protein, respectively, against the time of simulation (100 ns).

The RMSD of ligands within the protein's active site was described against the time of simulation (Figure 11). Most compounds showed stability inside the protein's active site during the simulation except for compounds **3b** and **3g**.

Compound **3a** moved around 3–4 Å from its original site and moved deeper inside the active site; the fluctuation at ∼55–57 and 92–95 ns is due to losing interaction with residue Glu166. Moreover, compound **3b** was still stable inside the active pocket till around 40 ns before it lost its interactions and entirely moved out of the active site. This may recommend a great conformational change within the examined protein due to the interaction with compound **3b** which may explain its superior antiviral activity^52^. Compounds **3c**, **3d**, **3e**, **3f**, and WR1 behave nearly in the same way as compound **3a**, and the compounds moved deeper inside the active site than their initial position by around 8, 3, 4, 6, and 4 Å, respectively. Compound **3g** was not stable; it started to fluctuate from the beginning of the simulation and moved by 4 Å from its original site, and at around 28 ns and moved further by 4 Å from its new position up to about 75 ns, where it lost its interaction and pushed out the active site.

##### 2.3.2.2. Histogram and heat map analyses

**Figure 12.**
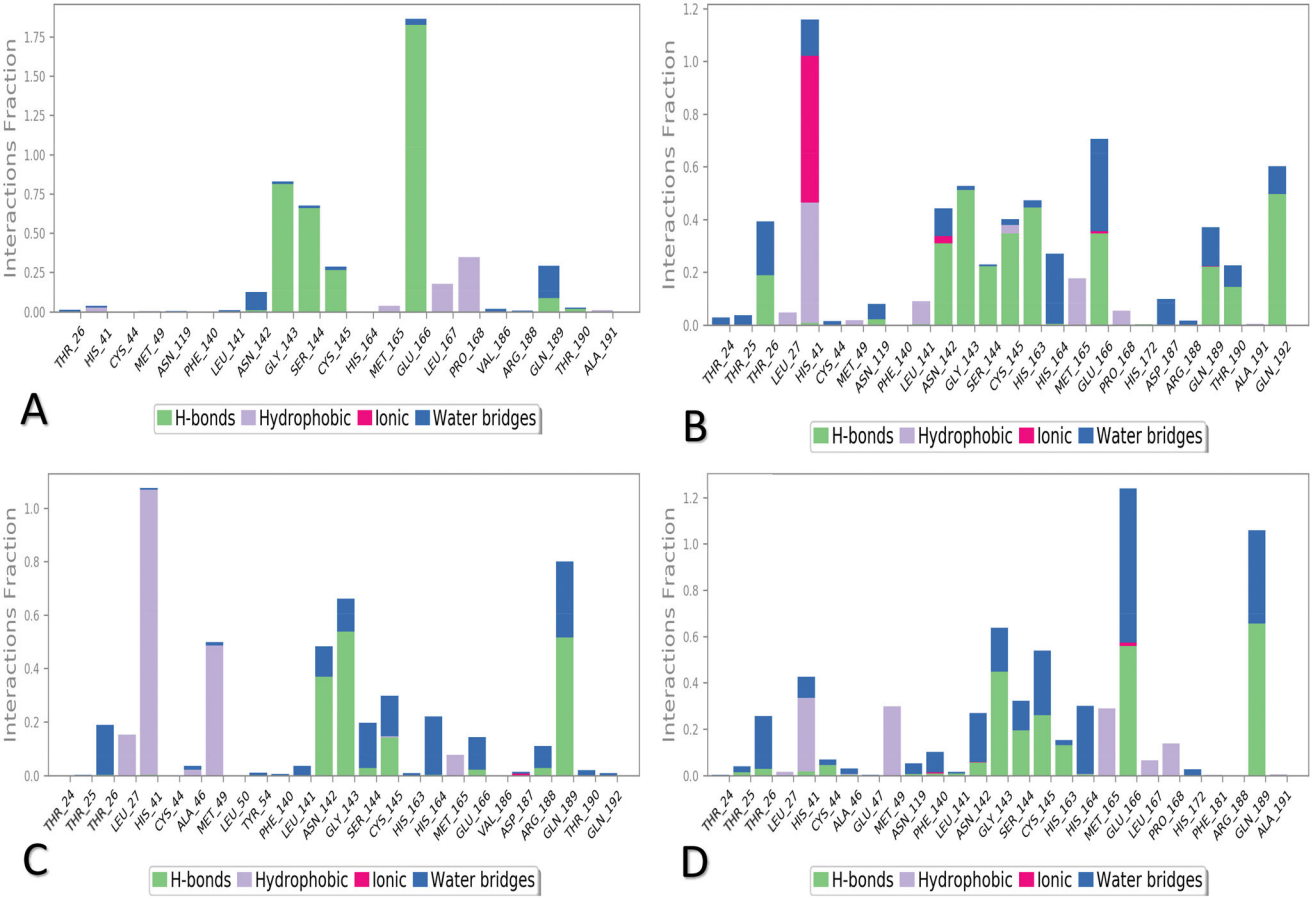
Histogram for the interactions between the tested ligand towards the SARS-CoV protein during the 100 ns of the simulation for (A) **3a**, (B) **3b**, (C) **3c**, and (D) WR1.

Histograms for the SARS-CoV protein-ligand contacts of the selected four complexes during the simulation time (100 ns) are described in Figure 12.

Regarding **3a**-complex, Glu166 contributed ∼90%, besides Gly143, Ser144, and Cys145 contributed (∼10–45%) of the interactions as H-bonding; however, Leu167 and Pro168 formed the hydrophobic interactions mainly. Also, Asn142 and Gln189 were the main members contributing to the H_2_O-bridges H-bonds, and also no ionic bonds were recorded. Obviously, Glu166 was the most participating amino acid in the interactions through hydrogen bonds (Figure 12(A)).

Moreover, Thr26, Asn142, Ser144, Gly143, Gln189, Cys145, Gln192, His163, Thr190, and Glu166 formed the main H-bonding for **3b**-complex; besides, His41 (∼35%), and Met165 amino acids formed the hydrophobic interactions. Ionic interactions were formed mainly through His41 (∼40%); and Glu166 and Thr26 amino acids formed mainly the water bridges hydrogen bonds. Notably, His41 amino acid was the most contributing one in the interactions through hydrophobic-, ionic-, and H_2_O bridges H- bonds (Figure 12(B)).

Furthermore, the histogram of **3c**-complex showed that Asn142, Gly143, and Gln189 amino acids formed >35% of the hydrogen bonds; whereas His41 (>90%), Met49 (⁓40%), and Leu27 contributed to the hydrophobic interactions mainly. Ionic interactions were only observed through a small contribution of Asp187 amino acid. Moreover, the H_2_O bridges H-bonds were formed through Thr26, His164, and Gln189 mainly. His41 amino acid was the principal amino acid that contributed to the binding fraction as well (Figure 12(C)).

Finally, the WR1-complex histogram -as a reference standard- represented that the principal amino acids for H-bonds were Gln189 (⁓55%), Gly143, and Glu166 (>35%); and the main members for hydrophobic interactions were His41 (∼30%), Met49, and Met165 amino acids. Also, the ionic interactions were only observed through small contributions of Phe140 and Glu166 amino acids; and Glu166 (>40%) and Gln189 amino acids formed mainly the water bridges hydrogen bonds. It was clear that both Glu166 and Gln189 were the most types that contributed to the binding fraction through H- and H_2_O bridges H- bonds (Figure 12(D)).

**Figure 13.**
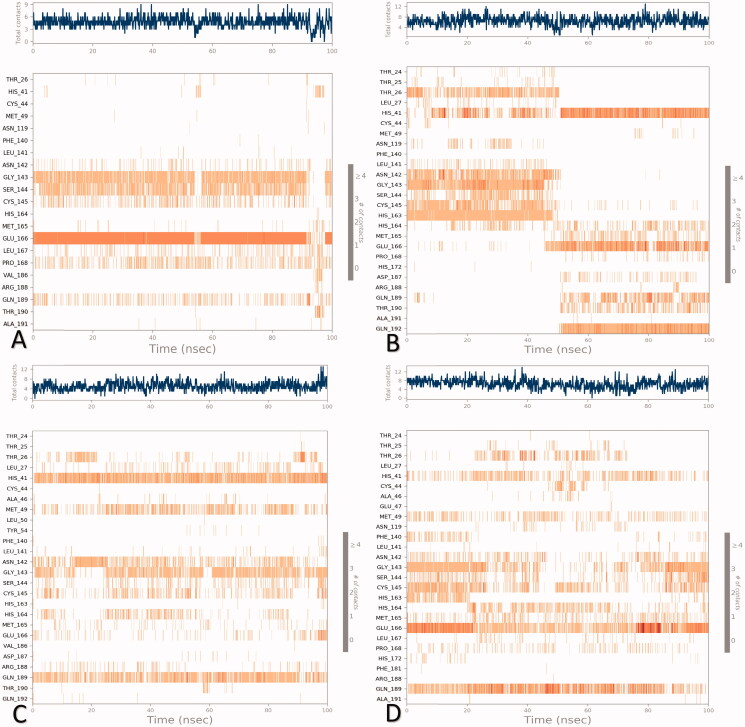
Heat map for SARS-CoV protein-ligand contacts all over the 100 ns of simulation for (A) **3a**, (B) **3b**, (C) **3c**, and (D) WR1.

The heat maps refer to the total number of contacts of **3a**, **3b**, **3c**, and WR1 within the SARS-CoV active pocket concerning the simulation time are depicted in Figure 13.

It was obvious that the principal interactions for **3a** within the SARS-CoV active site were through Glu166 (>90%), Gly143 (>50%), and Ser144 (>50%) amino acids all over the simulation time (Figure 13(A)). However, the binding residues for **3b** within the SARS-CoV active site were His41 (>95%) and Glu166 (>50%) amino acids throughout the 100 ns of simulation (Figure 13(B)). At the same time, His41 (>95%) and Gln189 (>70%) were the main amino acid residues for the interactions with **3c** within SARS-CoV binding pocket throughout the simulation time (Figure 13(C)). Furthermore, the main binding residues to WR1 were observed to be Glu166 (>90%) and Gln189 (>80%) at the time of simulation (Figure 13(D)). This concludes the great importance of Glu166, Gln189, and His41 amino acids for the interactions with the expected inhibitors within the binding pocket of SARS-CoV.

Moreover, the previously reported Glu166 residue to be critical in the ligand-binding inside the active pocket of SARS-CoV Mpro^17^ was used for distance measurements (Supplementary Data, Supplementary Figure 3). Besides, the histograms and heat maps for compounds **3d**, **3e**, and **3f** were provided in the Supplementary Data (Supplementary Figure 4).

##### 2.3.2.3. Analysis of ligand properties

**Figure 14.**
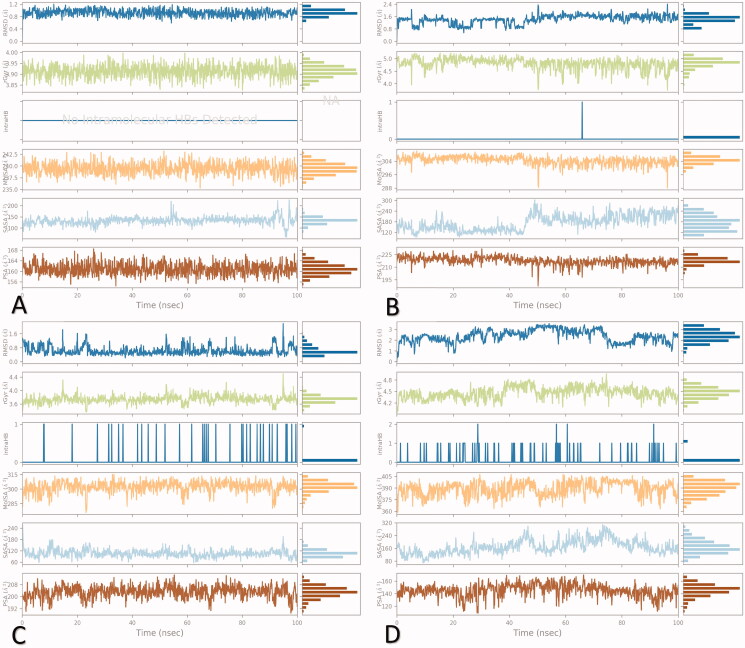
Ligand properties during the 100 ns of simulation for (A) **3a**, (B) **3b**, (C) **3c**, and (D) WR1.

Ligand properties include the RMSD, Intramolecular H-bonds (intraHB), Radius of Gyration (rGyr), Molecular Surface Area (MolSA), Polar Surface Area (PSA), and Solvent Accessible Surface Area (SASA), as depicted in Figure 14.

The RMSD and rGyr for **3a**-complex were observed to be within the range of (0.6–1.2) and (3.8–4) Å with equilibrium values around 0.9 and 3.92 Å, respectively. Also, no intraHB was observed during the 100 ns of simulation and the MolSA range was within (236–243.5 Å^2^) and showed small fluctuations during the 100 ns of simulation reaching an equilibrium at about 240 Å^2^. Moreover, the SASA was within the (50–200 Å^2^) range and showed fluctuations after 90 ns with an equilibrium around 140 Å^2^. Moreover, its PSA was between 156 and168 Å^2^ with the equilibrium at 161 Å^2^ (Figure 14(A)).

Furthermore, for the **3b**-complex, the RMSD was (0.8–2.4 Å) and achieved an equilibrium of around 1.6 Å. The rGyr was in between (4–5.5 Å) with an equilibrium around 4.8 Å. The intraHB appeared as a small band at about 65 ns only. Both MolSA and SASA were within the (288–312) and (100–300) Å^2^ range and showed equilibrium around 304 and 180 Å^2^, respectively. The SASA showed fluctuations at 45 ns and persisted up to the end and the PSA was within the (195–225 Å^2^) range with a small fluctuation at 50 ns (Figure 14(B)).

Furthermore, for **3c**-complex, the RMSD and rGyr were within the range of (0.6–1.6) and (3.4–4.4) Å with observed equilibrium values around 0.6 and 3.8 Å, respectively. Notably, the intraHB was observed through the 100 ns of simulation and increased in the second half as well. The MolSA fluctuated between (280 and 315 Å^2^) with an equilibrium at 305 Å^2^, while the SASA was within (60–240 Å^2^) where its fluctuations decreased after the beginning of the simulation with an equilibrium around 110 Å^2^. On the other hand, the PSA fluctuations were within the (190–212 Å^2^) range with the equilibrium at 204 Å^2^ (Figure 14(C)).

Finally, the co-crystallized inhibitor (WR1-complex) showed an RMSD within the (0.5–3.5 Å) range with large fluctuations all over the 100 ns of simulation and the equilibrium was at 2 Å. Also, the rGyr was within the range of (4–4.8 Å) with more fluctuations from 40 ns to the end of the 100 ns of simulation and the equilibrium was observed around 4.5 Å. Moreover, the intraHB appeared from the beginning until the end of the 100 ns. The MolSA fluctuations were within the (360–405 Å^2^) range and got their equilibrium at 395 Å^2^. At the same time, the SASA appeared as large fluctuations (80–320 Å^2^) along the simulation time with an equilibrium at 160 Å^2^. Furthermore, its PSA fluctuations were within the (100–180 Å^2^) range and achieved equilibrium at 150 Å^2^ (Figure 14(D)).

Based on the above, we can conclude that both compounds **3b** and **3c** were greatly similar to the WR1 inhibitor in the intraHB presence indicating corresponding similar binding behaviours. Also, the properties of their ligand were superior to those of WR1 which recommend a preferable binding affinity and consequently a promising intrinsic activity as expected.

#### 2.3.3. MM-GBSA calculations

The Coulomb, Hydrogen-bonding, Covalent-binding, Generalised Born electrostatic solvation, Lipophilic, and Van der Waals energies were calculated using the mean MM-GBSA binding energy supported by Schrodinger^30^^,^^53^. All the got results are depicted in Table 2.

**Table 2.** MM-GBSA energies (kcal/mol) for complexes (**3a**–**g** and WR1) of SARS-CoV.

Complex	ΔG Binding	Coulomb	Covalent	H-bond	Lipo	Bind Packing	Solv_GB	VdW	St. Dev.
**3a**	−42.31	−5.15	1.99	−1.87	−10.13	−1.96	5.67	−30.86	5.04
**3b**	−44.50	−12.90	2.72	−1.43	−8.36	−0.97	14.68	−38.23	5.95
**3c**	−45.65	−11.84	2.59	−1.00	−8.34	−0.94	13.45	−39.55	6.44
**3d**	−51.13	−9.99	−0.06	−1.37	−10.31	−2.46	11.04	−37.96	3.15
**3e**	−51.86	−9.17	3.09	−2.13	−12.63	−2.07	13.10	−42.04	5.50
**3f**	−48.41	−3.20	1.44	−1.09	−10.63	−1.32	14.91	−48.51	4.58
**3g**	−44.67	−14.43	2.64	−1.27	−9.57	−1.78	16.21	−36.45	6.66
WR1	−60.82	−27.28	1.95	−2.05	−14.64	−2.40	28.83	−45.23	7.07

Lipo: lipophilic energy; Solv_GB: generalised born electrostatic solvation energy; VdW: Van der Waals energy; St. Dev.: standard deviation.

As it can be seen from Table 2, the WR1 has the highest MM-GBSA binding energy of −60.82 kcal/mol. Compounds **3d** and **3e** showed similar binding energy of −51.13 and −51.86 Kcal/mol, respectively. **3e** also showed a similar H-bond energy and lipophilic energy to WR1. Other compounds have binding energies from −42 to −48 kcal/mol which is outstanding for these compounds' mechanism of action to be presented as potent SARS-CoV Mpro inhibitors. Notably, compound **3b** showed significant binding energy (−44.50 kcal/mol) relative to the co-crystallized WR1 inhibitor (−60.82 kcal/mol). On the other hand, it showed superior covalent binding energy (2.72) compared to the reference docked inhibitor with (1.95).

#### 2.3.4. Prediction of pharmacokinetic and physicochemical properties

The pharmacokinetic and physicochemical properties of the synthesised derivatives **3a–g** were described using SwissADME (the online web tool) as depicted in Table 3. Concerning their physicochemical properties, all of the synthesised compounds are from moderately soluble to soluble in water and thus much fewer concerns may be encountered in drug formulations. It was suggested that for any drug to be absorbed, it should be available at the absorption site in solution form^54^.

**Table 3.** Physicochemical and ADMET studies of the novel candidates **3a–g** and WR1.

		Investigated compounds							
		Comp **3a**	Comp **3b**	Comp **3c**	Comp **3d**	Comp **3e**	Comp **3f**	Comp **3g**	Comp WR1
Molecular properties	Molar Refractivity	70.12	83.83	88.63	98.25	113.12	100.14	124.98	108.00
	TPSA (A^z^)	116.05	154.38	154.38	154.38	154.38	145.59	170.17	104.73
	Log *P* o/w (WLOGP)	2.42	1.57	1.96	2.60	3.18	2.07	3.67	1.83
	Consensus Log *P* o/w	1.84	1.13	1.26	1.87	2.49	1.60	2.65	2.08
	Water solubility	Soluble	Soluble	Soluble	Soluble	Moderate	Soluble	Moderate	Soluble
Pharmacokinetics parameters	GI absorption	High	Low	Low	Low	Low	Low	Low	High
	BBB permeant	No	No	No	No	No	No	No	No
	P-gp substrate	No	No	No	No	No	No	No	Yes
	CYP1A2 inhibitor	Yes	No	No	Yes	No	No	No	No
	CYP2C19 inhibitor	Yes	Yes	Yes	Yes	Yes	Yes	Yes	No
	CYP2C9 inhibitor	No	No	No	Yes	Yes	Yes	Yes	No
	CYP2D6 inhibitor	No	No	No	No	No	No	No	Yes
	CYP3A4 inhibitor	No	No	No	Yes	Yes	Yes	Yes	No
Drug/lead likeness	Drug likeness (Lipiniski)	Yes	Yes	Yes	Yes	Yes	Yes	Yes	Yes
	Lead likeness	Yes	No	No	No	No	No	No	No
Toxicity parameters	Ames toxicity	Yes	No	No	No	No	Yes	No	No
	Max. tolerated dose (log mg/kg/day)	0.438	0.478	0.402	0.333	−0.427	−0.008	−0.066	0.186
	hERG I inhibitor	No	No	No	No	No	No	No	No
	hERG II inhibitor	No	No	No	No	No	No	Yes	Yes
	Oral rat acute toxicity (LD50) (mol/kg)	2.826	2.836	2.833	2.957	3.726	2.897	3.589	2.01
	Oral rat chronic toxicity (LOAEL) (log mg/kg_bw/day)	1.506	1.26	1.392	1.67	1.56	1.032	1.681	0.79
	Hepatotoxicity	No	Yes	Yes	Yes	Yes	Yes	Yes	Yes
	Minnow toxicity (log mM)	0.901	2.616	2.754	2.035	1.841	2.696	2.25	4.222

Besides, concerning the ADME results, except for compounds **3a** and WR1, the other synthesised compounds attain unfortunately low GIT absorption due to their poor lipophilicity. So oral route may not be suitable for these compounds if administered in their current form. All of the synthesised compounds do not cross the blood-brain barrier (BBB), hence these compounds may not encounter CNS side effects^55^. Fortunately, all of the synthesised compounds are not substrates for P-glycoprotein (Pgp-), so they may be not susceptible to this efflux mechanism. Besides, compounds **3a**–**c** exhibit less inhibiting power towards the most common hepatic metabolising enzymes (CYP 1A2, CYP3A4, CYP2C9, CYP2C19, and CYP2D6) among other synthesised compounds. Moreover, Lipinski’s rule^56^ is not violated by all synthesised compounds, so assuring their advantage as drug members. Notably, compound **3a** may be utilised as a lead compound for future optimizations.

Moreover, the toxicity of the synthesised candidates could be predicted using the pkCSM descriptors algorithm protocol. Except for compounds **3a,f**, all other candidates do not experience Ames toxicity, and so they could not be considered mutagenic agents^57^. Besides, all the synthesised candidates do not exhibit a cardiotoxic effect since they are non-inhibitors of *h*ERG I^58^. Additionally, except for compound **3f**, all of the synthesised derivatives could be regarded as non-inhibitors of *h*ERG II, hence the cardiac arrhythmia threat may be avoided^59^. Also, compound **3a** is non-hepatotoxic. Finally, compounds **3b,c,f** show feasible tolerability due to their oral rat chronic toxicity (*in silico*) relative to lower values.

### 2.4. Structure–activity relationship (SAR) study

**Figure 15.**
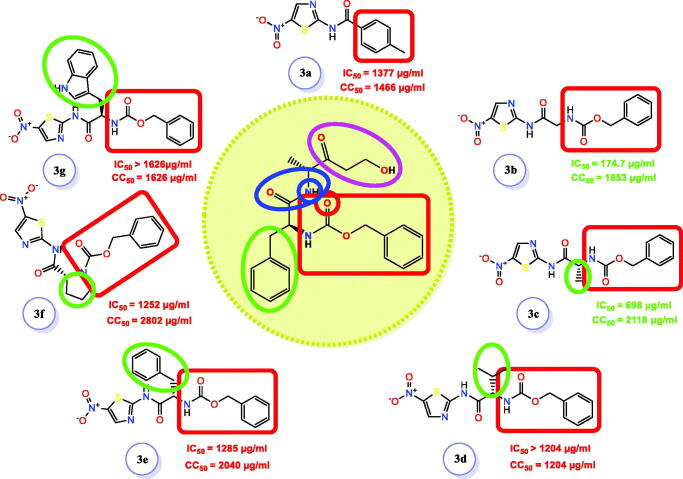
SAR studies of the newly designed targets (**3a–g**) as SARS-CoV-2 Mpro inhibitors. The red rectangle refers to the H-bond acceptor moiety and the green circle refers to the moiety that fits into the hydrophobic groove.

According to *in vitro* results, acylation of amino thiazole with amino acid enhanced antiviral activity. Notably, the activity was inversely proportional to the size of the substituent at the α-position (Figure 15)^60^. So, a bulky substituent at α-position diminished the activity of synthesised compounds against SARS-CoV. However, direct acylation of the aminothiazole with an aromatic ring didn’t improve the antiviral activity.

Therefore, based on both the *in vitro* (Figures 5 and 6) and the *in silico* (Figures 9 and 11, and Table 1) results, we can conclude the following interesting points describing the recommended structure-activity relationship (SAR) of the examined candidates (**3a–g**) as depicted in Figure 15:

- Compound **3b** with no hydrophobic side chain (either aliphatic or aromatic) showed the best anti-SARS-CoV-2 (174.7 µg/mL) and almost the SARS-CoV-2 Mpro inhibition (5.12 µg/mL) activities as well. Its docking score (−6.94 kcal/mol) was very promising compared to other candidates and its binding mode was nearly similar to that of the native co-crystallized WR1. This may be attributed to its good penetration throughout the cells of SARS-CoV-2.
- Compound **3d** designed with the isopropyl hydrophobic side chain showed a superior docking score (−7.01 kcal/mol) compared to that of WR1 (−6.52 kcal/mol). Also, its ΔG binding energy calculated from the MM-GBSA (−51.13 kcal/mol) was promising compared to that of WR1 (−60.82 kcal/mol). The MD simulations of compound **3d** showed that it moved deeper inside the active site of SARS-CoV than its initial position by around 3 Å indicating a stable behaviour as well. However, its SARS-CoV-2 inhibitory activity was higher than its corresponding CC_50_ value with a weak SARS-CoV-2 Mpro inhibition (88.84 µg/mL) performed through a cell-based induced assay. This may be explained by expecting the very poor penetration of compound **3d** throughout the viral cells which inversely affected its antiviral activity. Therefore, a suitable formulation for compound **3d** is required soon to confirm the recommended hypothesis.
- Compound **3a** with the smallest size showed a weak activity against SARS-CoV-2 (1377 µg/mL) but a highly promising SARS-CoV-2 Mpro inhibition (4.67 µg/mL). This indicates that the Mpro inhibitory activity is inversely proportional to the size of the substituent at the α-position.
- Compound **3c** having a simple methyl hydrophobic side chain showed superior SARS-CoV-2 inhibitory activity (698 µg/mL) with a promising Mpro inhibition (11.90 µg/mL). It showed very significant values of the binding score and ΔG binding energy (−6.07 and −45.65 kcal/mol, respectively) with almost the same binding mode as the native co-crystallized WR1 inhibitor.
- Compound **3g** with the largest hydrophobic indole side chain was not stable during the MD simulations. It started to fluctuate from the beginning of the simulation and moved away from its original position till it lost its interaction and was pushed out of the active site. At the same time, its SARS-CoV-2 inhibitory activity was higher than its corresponding CC_50_ value and showed a very weak inhibition towards the SARS-CoV-2 Mpro (51.37 µg/mL). This confirms again that the Mpro inhibition is inversely related to the size of the substituent at the α-position.
- Both compounds **3e** and **3f** with benzyl and pyrrolidine side chains were observed to be weak members against SARS-CoV-2 with IC_50_ values of 1285 and 1252 µg/mL, respectively. However, their SARS-CoV-2 Mpro inhibitory activities were moderate with IC_50_ values of 16.57 and 22.37 µg/mL, respectively. This may explain their good docking scores, binding modes, ΔG binding energies, and MD results towards SARS-CoV Mpro as a target receptor.
- All the designed derivatives bound both Glu166 and Cys145 amino acids which are crucial for the inhibition of the SARS-CoV Mpro active site.

Furthermore, a multiple linear regression model was established to assess the correlation between the two independent variables (anticipated Log *P* and docking score) and the dependent variable (IC_50_ values) as shown in Table 4. It was revealed that *R*^2^ was 0.49. Thus, in other words, we can conclude that nearly 49% of the IC_50_ values’ variability could be elucidated by the independent Log *P* and docking scores entire set.

**Table 4.** The IC_50_ values (µg/mL), log*P*, and docking scores of the examined candidates for the construction of the multiple linear regression model.

Comp.	IC_50_	S score	Log *P*
**3a**	4.66	−5.43	2.42
**3b**	5.11	−6.94	1.57
**3c**	11.90	−6.07	1.96
**3d**	88.84	−7.01	2.60
**3e**	16.57	−6.44	3.18
**3f**	22.37	−6.59	2.07
**3g**	51.37	−6.80	3.67

## 3. Conclusion

Owing to COVID-19 global expansion and overwhelming spread with the rising death toll, scientists and researchers are committed to developing new effective drugs as fast as possible. So, in this presented work, a novel wave of *N*-(5-nitrothiazol-2-yl)-carboxamido derivatives (**3a–g**) was designed and chemically synthesised based on the fundamental pharmacophoric features of the co-crystallized inhibitor WR1 of SARS-CoV. Compound **3b** was the superior anti-SARS-CoV-2 candidate with an IC_50_ of 174.7 µg/mL. Moreover, the drug candidates **3a**, **3b**, and **3c** experienced potential SARS-CoV-2 Mpro inhibition with IC_50_ of 4.67, 5.12, and 11.90 µg/mL, respectively. Hence, the attained results extremely assured our designed rationale and comply with the attained computational insights using molecular docking and dynamics simulations which declared the strong anticipated activities for these drug candidates. The promising compounds **3a**, **3b**, and **3c** displayed binding interactions of −5.43, −6.94, and −6.07 kcal/mol, respectively. Furthermore, the presented work shed light on the SAR of the synthesised derivatives **3a–g** pointing out a structural modification that could enhance activity against COVID-19 for future design. Obviously, the activity was inversely proportional to the size of the substituent at the α-position. So, a bulky substituent at α-position diminished the activity of synthesised compounds against SARS-CoV. Therefore, based on the above, compound **3b** with no hydrophobic side chain (either aliphatic or aromatic) showed the best anti-SARS-CoV-2 (174.7 µg/mL) and almost the SARS-CoV-2 Mpro inhibition (5.12 µg/mL) activities as well. Its docking score (−6.94 kcal/mol) was very promising compared to other candidates and its binding mode was nearly similar to that of the native co-crystallized WR1. This may be attributed to its good penetration throughout the cells of SARS-CoV-2. Finally, most investigated compounds, particularly compound **3b**, showed feasible tolerability in ADMET studies.

## 4. Materials and methods

### 4.1. Chemistry

#### 4.1.1. General

All materials were purchased from commercial suppliers and used with no extra purification. The final compounds' purities were elucidated by tandem mass spectrometry (LC/MS) using a gradient elution system (acetonitrile/water 5/95/95/5, 5 min, 0.05% formic acid) on Ascentis Express Peptide C18 column, and UV detection (254 nm). The final compounds' purities were 95% or greater. A Bruker NMR 400 MHz Avance III spectrometer operating at 100 MHz for ^13^C NMR and 400 MHz for ^1^H NMR was utilised for NMR spectra recording. Chemical shifts are given relative to tetramethylsilane (TMS) in part per million (ppm), and coupling constants *J* are given in Hertz. HPLC-HRMS analyses were carried out using Agilent (Santa Clara, CA) 1200 series binary pump (G1312B), and columns waters XTerra MS C_18_ (3.5 um; 2.1 × 150 mm) + Phenomenex C_18_ security guard column (2 × 4 mm) on gradient elution mobile phase using 0.2% acetic acid in H_2_O/methanol; wavelength = 254 nm. Elemental analyses were established at Microanalytical Centre, Faculty of Science, Cairo University, Egypt using Manual Elemental Analyser Heraeus (Germany) and Automatic Elemental Analyser CHN Model 2400 Perkin Elmer (USA).

#### 4.1.2. General procedure for the synthesis of compounds 3a–3g

*N*-Acyl benzotriazoles (1 equiv, 0.2 mmol) were added to a stirred solution of 5-nitrothiazol-2-amine (1.1 equiv, 0.22 mmol) and triethylamine (1.1 equiv, 0.22 mmol) in acetonitrile (4 ml). The reaction mixture was stirred for 1 h. The solvent was evaporated, and the residue was acidified with HCl (2 N). The precipitated solid was filtered, washed with HCl (2 N), water, and dried to obtain the desired products. All the NMR analysis data of the target compounds (**3a–g**) was added to the Supplementary Data (SI1).

##### 4.1.2.1. 4-Methyl-N-(5-nitrothiazol-2-yl)benzamide (3a)


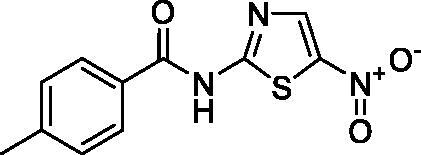
 Yellow microcrystals (96%). ^1^H NMR (500 MHz, DMSO*-*d_6_) *δ* 3.47 (s, 1H), 8.69 (s, 1H), 8.05–8.03 (m, 2H), 7.38 (d, *J*= 8.0 Hz, 2H), 2.40 (s, 3H); ^13^C NMR (125 MHz, DMSO-d_6_) *δ* 166.7, 163.2, 144.5, 143.1, 142.5, 129.8, 129.1, 128.4, 21.6. LC/MS *m/z*: 264 [M + H^+^]. Anal. Calcd. for C_11_H_9_N_3_O_3_S: C, 50.18; H, 3.45; N, 15.96. Found: C, 50.22; H, 3.37; N, 15.93.

##### 4.1.2.2. Benzyl (2-((5-nitrothiazol-2-yl)amino)-2-oxoethyl)carbamate (3b)


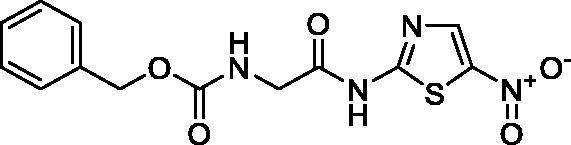
 Yellow microcrystals (92%). ^1^H NMR (500 MHz, DMSO-d_6_) *δ* 13.19 (s, 1H), 8.61 (s, 1H), 7.76 (t, *J*= 5.1 Hz, 1H), 7.35–7.29 (m, 5H), 5.04 (s, 3H), 4.01 (d, *J*= 4.4 Hz, 2H); ^13^C NMR (125 MHz, DMSO-d_6_) *δ* 170.7, 162.0, 157.0, 143.2, 137.4, 137.3, 128.8, 128.3, 128.3, 66.2, 44.0. LC/MS *m/z*: 337 [M + H^+^]. Anal. Calcd. for C_13_H_12_N_4_O_5_S: C, 46.43; H, 3.60; N, 16.66. Found: C, 46.48; H, 3.55; N, 16.71.

##### 4.1.2.3. Benzyl (S)-(1-((5-nitrothiazol-2-yl)amino)-1-oxopropan-2-yl)carbamate (3c)


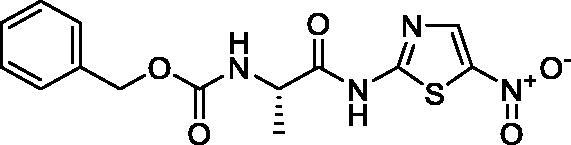
 Yellow microcrystals (94%). ^1^H NMR (500 MHz, DMSO-d_6_) *δ* 13.25 (s, 1H), 8.63 (s, 1H), 7.90 (d, *J*= 6.2 Hz, 1H), 7.41–7.11 (m, 5H), 5.01 (s, 2H), 4.33 (s, 1H), 1.30 (d, *J*= 6.6 Hz, 3H); ^13^C NMR (125 MHz, DMSO-d_6_) *δ* 174.1, 162.1, 156.3, 143.1, 142.5, 137.2, 128.8, 128.3, 128.3, 66.1, 50.6, 17.6. LC/MS *m/z*: 351 [M + H^+^]. Anal. Calcd. for C_14_H_14_N_4_O_5_S: C, 48.00; H, 4.03; N, 15.99. Found: C, 48.11; H, 4.09; N, 15.88.

##### 4.1.2.4. Benzyl (S)-(3-methyl-1-((5-nitrothiazol-2-yl)amino)-1-oxobutan-2-yl)carbamate (3d)


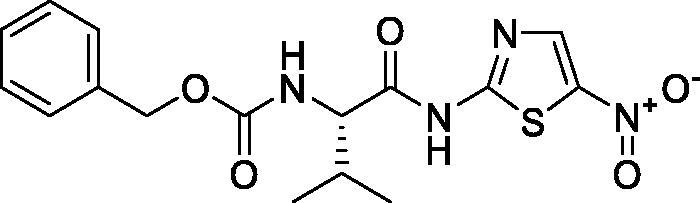
 Yellow microcrystals (91%). ^1^H NMR (500 MHz, DMSO-d_6_) *δ* 13.27 (s, 1H), 8.60 (s, 1H), 7.81 (d, *J*= 7.6 Hz, 1H), 7.42–7.13 (m, 5H), 5.00 (s, 2H), 4.15 (t, *J*= 7.3 Hz, 1H), 2.05–1.99 (m, *J*= 13.5, 6.5 Hz, 1H), 0.86 (t, *J*= 6.4 Hz, 6H); ^13^C NMR (125 MHz, DMSO-d_6_) *δ* 173.2, 161.6, 156.8, 143.1, 142.5, 137.2, 128.8, 128.3, 128.3, 66.2, 60.7, 30.3, 19.4, 18.7. LC/MS *m/z*: 379 [M + H^+^]. Anal. Calcd. for C_16_H_18_N_4_O_5_S: C, 50.79; H, 4.79; N, 14.81. Found: C, 50.87; H, 4.73; N, 14.89.

##### 4.1.2.5. Benzyl (S)-(1-((5-nitrothiazol-2-yl)amino)-1-oxo-3-phenylpropan-2-yl)carbamate (3e)


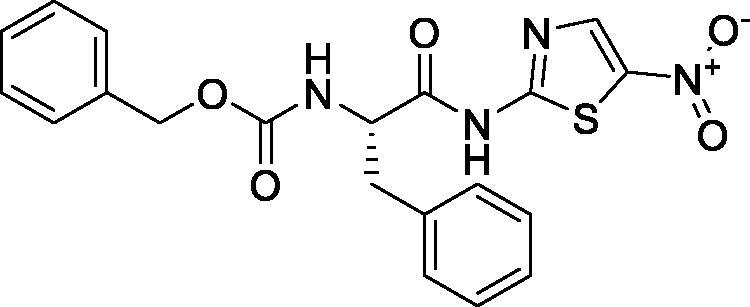
 Brownish yellow microcrystals (95%). ^1^H NMR (500 MHz, DMSO-d_6_) *δ* 13.27 13.43 (s, 1H), 8.62 (s, 1H), 7.97 (d, *J*= 7.8 Hz, 1H), 7.39–7.12 (m, 10H), 4.93 (s, 2H), 4.57–4.50 (m, 1H), 3.05 (dd, *J*= 13.6, 3.9 Hz, 1H), 2.87–2.76 (m, 1H); ^13^C NMR (125 MHz, DMSO-d_6_) *δ* 173.1, 161.9, 156.5, 143.1, 142.5, 137.5, 137.1, 129.7, 128.8, 128.6, 128.3, 128.1, 127.1, 66.0, 56.8, 37.0. LC/MS *m/z*: 427 [M + H^+^]. Anal. Calcd. for C_20_H_18_N_4_O_5_S: C, 56.33; H, 4.25; N, 13.14. Found: C, 56.41; H, 4.29; N, 13.21.

##### 4.1.2.6. Benzyl (S)-2-((5-nitrothiazol-2-yl)carbamoyl)pyrrolidine-1-carboxylate (3f)


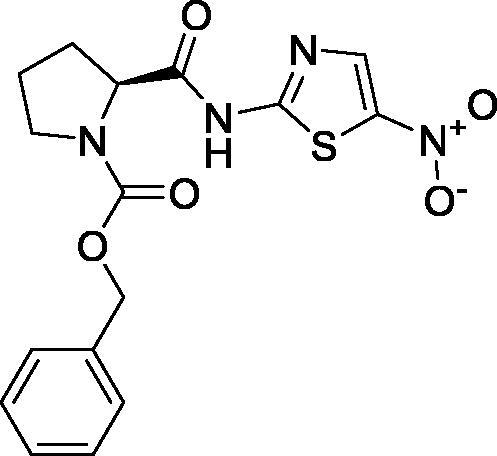
 Yellow microcrystals (93%). ^1^H NMR (500 MHz, DMSO-d_6_) *δ* 13.29 (s, 1H), 8.59 (s, 1H), 7.57–6.91 (m, 5H), 5.13–4.81 (m, 2H), 4.56–4.41 (m, 1H), 3.57–3.35 (m, 2H), 2.31–2.14 (m, 1H), 1.99–1.70 (m, 3H); ^13^C NMR (125 MHz, DMSO-d_6_) *δ* 173.4, 173.1, 161.9, 154.5, 153.7, 143.1, 143.0, 142.5, 137.2, 136.8, 128.8, 128.5, 128.3, 128.1, 128.0, 127.7, 66.7, 60.1, 59.6, 47.5, 47.0, 31.3, 30.3, 24.5, 23.7. LC/MS *m/z*: 377 [M + H^+^]. Anal. Calcd. for C_16_H_16_N_4_O_5_S: C, 51.06; H, 4.28; N, 14.89. Found: C, 51.13; H, 4.22; N, 14.75.

##### 4.1.2.7. Benzyl (S)-(3-(1H-indol-3-yl)-1-((5-nitrothiazol-2-yl)amino)-1-oxopropan-2-yl)carbamate (3g)


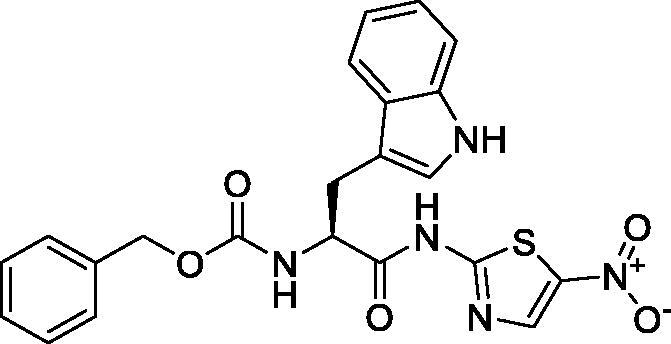
 Brown microcrystals (90%). ^1^H NMR (500 MHz, DMSO-d_6_) *δ* 13.46 (s, 1H), 10.84 (s, 1H), 8.60 (s, 1H), 7.87 (d, *J*= 7.2 Hz, 1H), 7.68 (d, *J*= 7.8 Hz, 1H), 7.44–7.07 (m, 7H), 7.02 (t, *J*= 7.3 Hz, 1H), 6.93 (t, *J*= 7.3 Hz, 1H), 4.93 (s, 2H), 4.51–4.56 (m, 1H), 3.17 (dd, *J*= 14.4, 5.0 Hz, 1H), 3.00 (dd, *J*= 13.7, 9.8 Hz, 1H); ^13^C NMR (125 MHz, DMSO-d_6_) *δ* 173.6, 162.0, 156.4, 143.1, 142.4, 137.1, 136.5, 128.8, 128.3, 128.2, 127.4, 124.8, 121.4, 119.0, 118.7, 111.8, 109.3, 66.1, 56.0, 27.7. LC/MS *m/z*: 466 [M + H^+^]. Anal. Calcd. for C_22_H_16_N_5_O_5_S: C, 56.77; H, 4.11; N, 15.05. Found: C, 56.84; H, 4.16; N, 15.02.

### 4.2. *In vitro* studies

#### 4.2.1. MTT assay

It was performed to calculate the newly synthesised candidates’ minimum concentrations that cause 50% toxicity to the cells (CC_50_). First, the newly synthesised derivatives were dissolved in ddH_2_O with 10% DMSO and then diluted with Dulbecco's Modified Eagle's Medium (DMEM) to the desired concentrations. The MTT assay method was performed with minor changes using VERO-E6 cells (ready for the virus propagation) to be applied in other experiments. The complete methodology was elucidated in the Supplementary Data (SI2).

#### 4.2.2. Inhibitory concentration 50 (IC_50_)

The IC_50_ for each examined compound (**3a–g**) which is equivalent to the minimum concentration to inhibit the virus infectivity by 50% compared to the virus control was calculated^61^. The full methodology was depicted in the Supplementary Data (SI3).

#### 4.2.3. SARS-CoV-2 Mpro assay (cell-based)

The Mpro activity was investigated using the 3CL Protease Assay Kit. The applied protocol and methodology were depicted in the Supplementary Data (SI4). Herein, the present assay was established to assess the newly synthesised candidates (**3a–g**) inhibitory effects on the SARS-CoV-2 Mpro enzyme as a recommended mechanism of action.

### 4.3. In silico studies

#### 4.3.1. Docking studies

The activity of synthesised derivatives (**3a–g**) against SARS-CoV Mpro, was investigated via molecular docking employing the MOE 2019 suite^62–65^. It was utilised to reveal the interactions of the aforementioned synthesised candidates towards SARS-CoV Mpro. Thereby, molecular docking was carried out to rationalise the mechanism of action for the synthesised derivatives as SARS-CoV Mpro inhibitors^66^.

##### 4.3.1.1. Preparation of the synthesized candidates 3a–g

The synthesised candidates were chemically drawn by PerkinElmer ChemOffice Suite 2019 version 19.0.0.22 and then prepared for docking as described in the default procedure^67–72^. The synthesised derivatives (**3a–g**) and the co-crystallized WR1 inhibitor were inserted into the same database (MDB file) and saved to be ready for SARS-CoV Mpro docking.

##### 4.3.1.2. Preparation of SARS-CoV Mpro receptor

The X-ray structure of SARS-CoV Mpro was obtained from the protein data bank online web (PDB entry: 2OP9)^41^. The target receptor was protonated, corrected for errors, and minimised energetically to be prepared for docking as discussed in detail^73–78^.

##### 4.3.1.3. Docking of the synthesized candidates to SARS-CoV Mpro target

The docking step was carried out and the docking protocol (general) was utilised to comply with the previously described methodologies^79–84^ to investigate poses with the most acceptable RMSD, scores, and interactions^85–88^.

#### 4.3.2. Molecular dynamics (MD) simulations

The desmond package of Schrödinger LLC^89^ was used to apply the MD simulations^90^^,^^91^. Moreover, the Schrodinger thermal_mmgbsa.py python script was used to measure the MM-GBSA energies for all examined complexes^29^^,^^92^^,^^93^. The full MD methodology was described in the Supplementary Data (SI5).

#### 4.3.3. MM-GBSA calculations

The Schrodinger thermal_mmgbsa.py python script was used to perform the average MM-GBSA binding energies^30^^,^^53^. Also, the Coulomb, Covalent-binding, Hydrogen-bonding, Generalised Born electrostatic solvation, Lipophilic, and Van der Waals energies were calculated. The methodology was depicted in the Supplementary Data (SI6).

#### 4.3.4. Prediction of pharmacokinetic and physicochemical properties

The pharmacokinetic and physicochemical investigation is an outstanding step in identifying novel candidates from a hit to a drug^94–96^. So, the Swiss Institute of Bioinformatics (SIB) supplies the free Swiss ADME evaluating the physicochemical, pharmacokinetic, and ADME parameters of the synthesised candidates could be predicted as well. Chemical structures of the synthesised derivatives (**3a–g**) and the co-crystallized ligand WR1 were transformed to SMILES, then submitted for further calculations^97^^,^^98^. Moreover, the toxicity features of the synthesised candidates were evaluated employing the pkCSM protocol^99^^,^^100^.

## 5. Statistical analysis

The results were represented as mean ± SD. One-way analysis of variance (ANOVA) followed by a Tukey–Kramer multiple comparison test. Then, the Kruskal-Wallis test followed by a Dunn's multiple comparison test was used for statistical comparison of parametric and nonparametric data, respectively.

## Supplementary Material

Supplemental Material
