# Supplementary material for: Ligand-based design, synthesis, computational insights, and in vitro studies of novel N-(5-Nitrothiazol-2-yl)-carboxamido derivatives as potent inhibitors of SARS-CoV-2 main protease
Source: J Enzyme Inhib Med Chem. 2022 Jul 31;37(1):2112–32. doi: 10.1080/14756366.2022.2105322 (PMC9344964; doi:10.1080/14756366.2022.2105322)
Supplement: Supplemental Material [file IENZ_A_2105322_SM1721.pdf]

Ligand-Based Design, Synthesis, Computational Insights, and *In Vitro* Studies of Novel *N*-(5-Nitrothiazol-2-yl)-Carboxamido Derivatives as Potent Inhibitors of SARS-CoV-2 Main Protease

Mohamed Elagawany[a]*, Ayman Abo Elmaaty[b], Ahmed Mostafa[c,d], Noura M. Abo Shama[c], Eman Y. Santali[e], Bahaa Elgendy[f,g], and Ahmed A. Al-Karmalawy[h]*

[a] Department of Pharmaceutical Chemistry, Faculty of Pharmacy, Damanhour University, Damanhour, Egypt.

[b] Department of Medicinal Chemistry, Faculty of Pharmacy, Port Said University, Port Said 42526, Egypt.

[c] Center of Scientific Excellence for Influenza Viruses, National Research Centre, Dokki, 12622, Cairo, Egypt.

[d] Institute of Medical Microbiology, German Center for Infection Research (DZIF), Justus-Liebig University Giessen, 35392 Giessen, Germany.

[e] Department of Pharmaceutical Chemistry, College of Pharmacy, Taif University, P.O. Box 11099, Taif 21944, Saudi Arabia.

[f] Center for Clinical Pharmacology, Washington University School of Medicine and University of Health Sciences, St. Louis, MO 63110, USA.

[g] Chemistry Department, Faculty of Science, Benha University, Benha 13518, Egypt.

[h] Department of Pharmaceutical Medicinal Chemistry, Faculty of Pharmacy, Horus University-Egypt, New Damietta 34518, Egypt.

* Corresponding authors:

Ahmed A. Al-Karmalawy: Email: akarmalawy@horus.edu.eg

ORCID: 0000-0002-8173-6073

Mohamed Elagawany: Email: elagawany@pharm.dmu.edu.eg

ORCID: 000-0002-1597-5324

Supplementary data

Figure SI 1. The RMSF values of the studied 2OP9 protein.

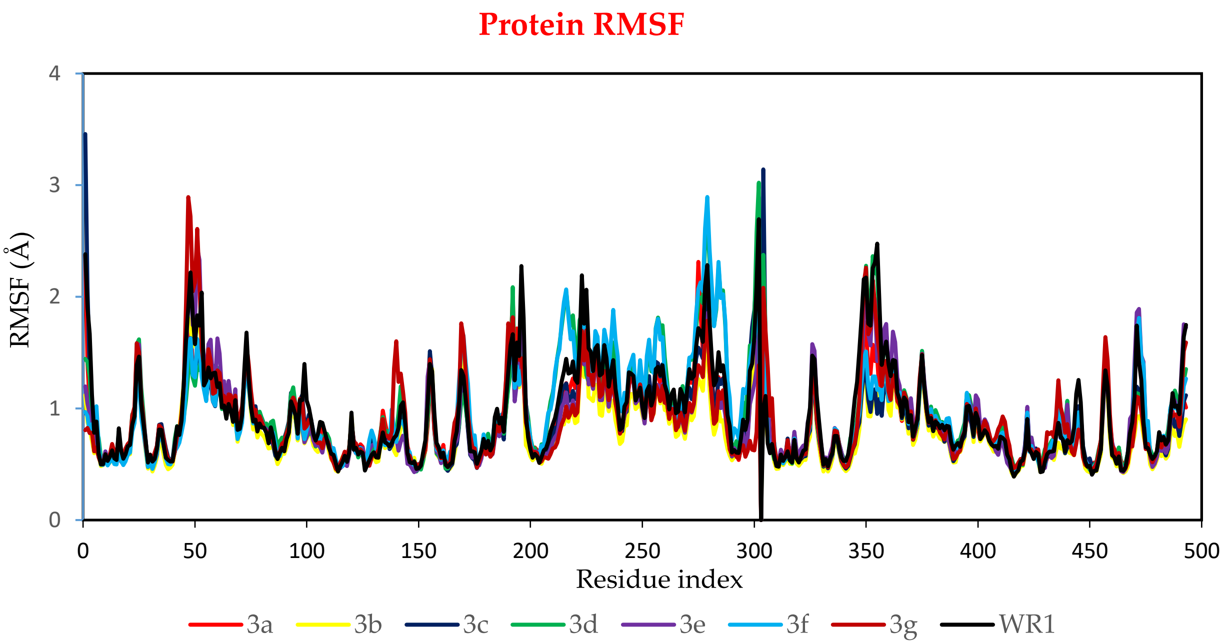

Figure SI 2. Snapshots at 0 ns, 50ns, and 100 ns of simulation time for 3a-2OP9 (**A**), 3b-2OP9 (**B**), 3c-2OP9 (**C**), and WR1-2OP9 (**D**).

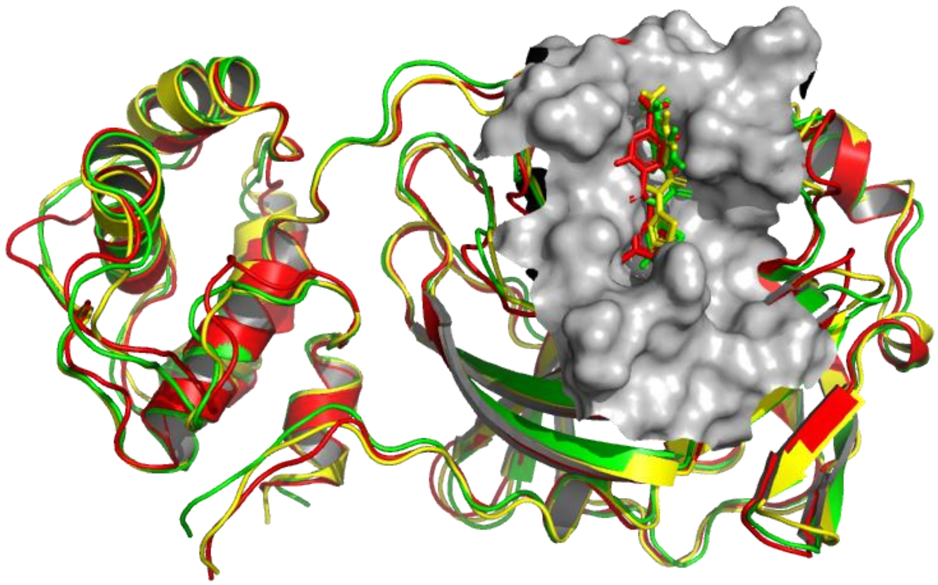

(**A**) 3a-2OP9 position at 0ns (red), 50ns (yellow) and 100ns (green) of simulation time.

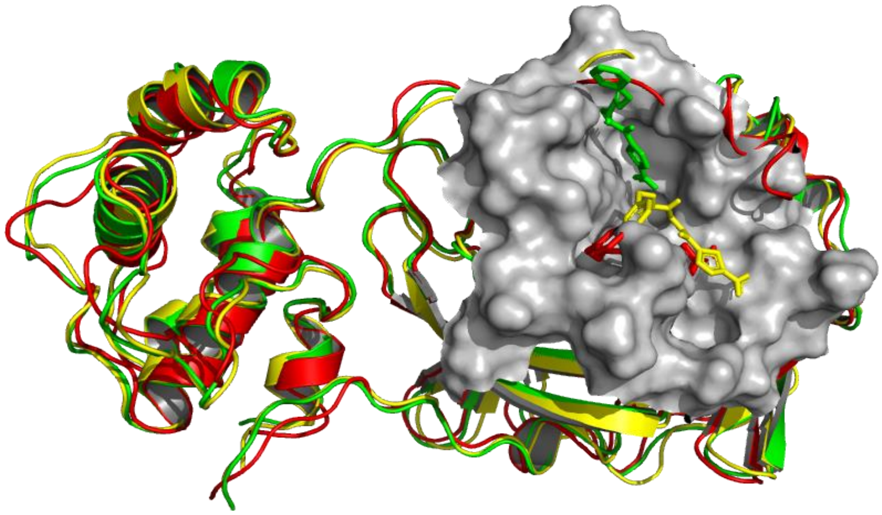

(**B**) 3b-2OP9 position at 0ns (red), 50ns (yellow) and 100ns (green) of simulation time.

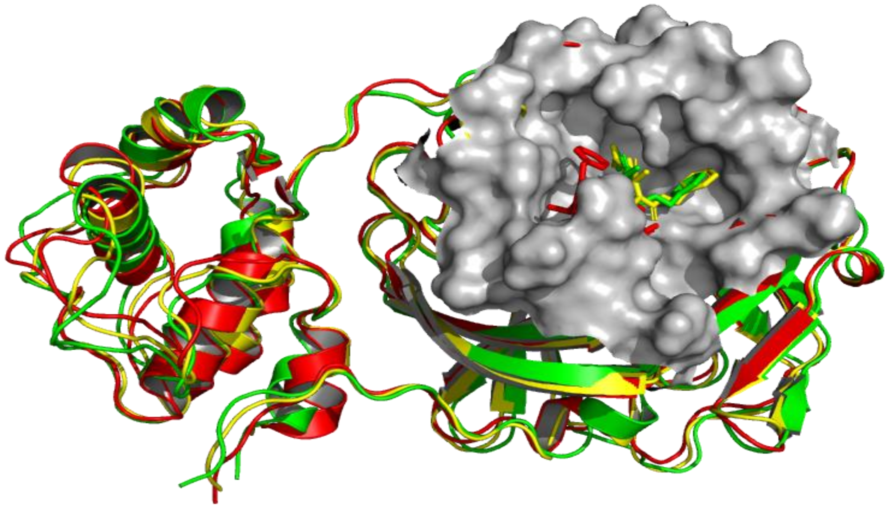

(**C**) 3c-2OP9 position at 0ns (red), 50ns (yellow) and 100ns (green) of simulation time.

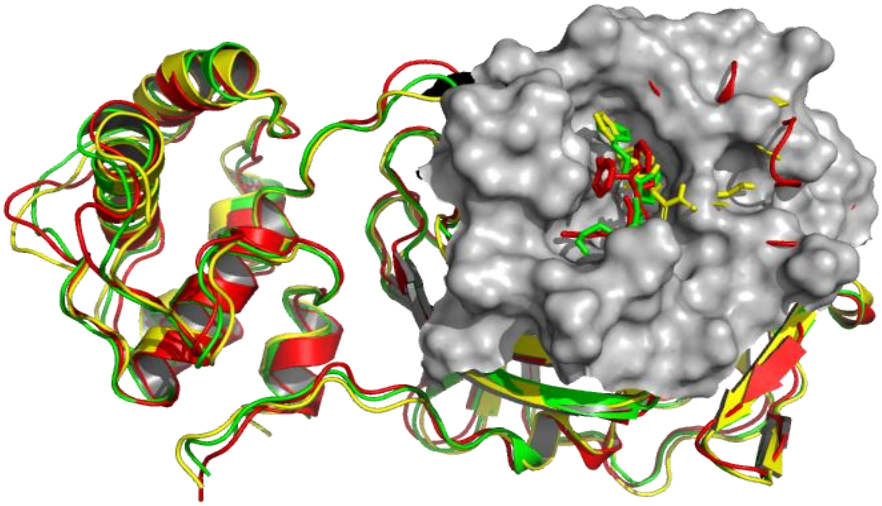

(**D**) WR1-2OP9 position at 0ns (red), 50ns (yellow) and 100ns (green) of simulation time.

Figure SI 3. Distance between center of mass of ligands and Glu166.

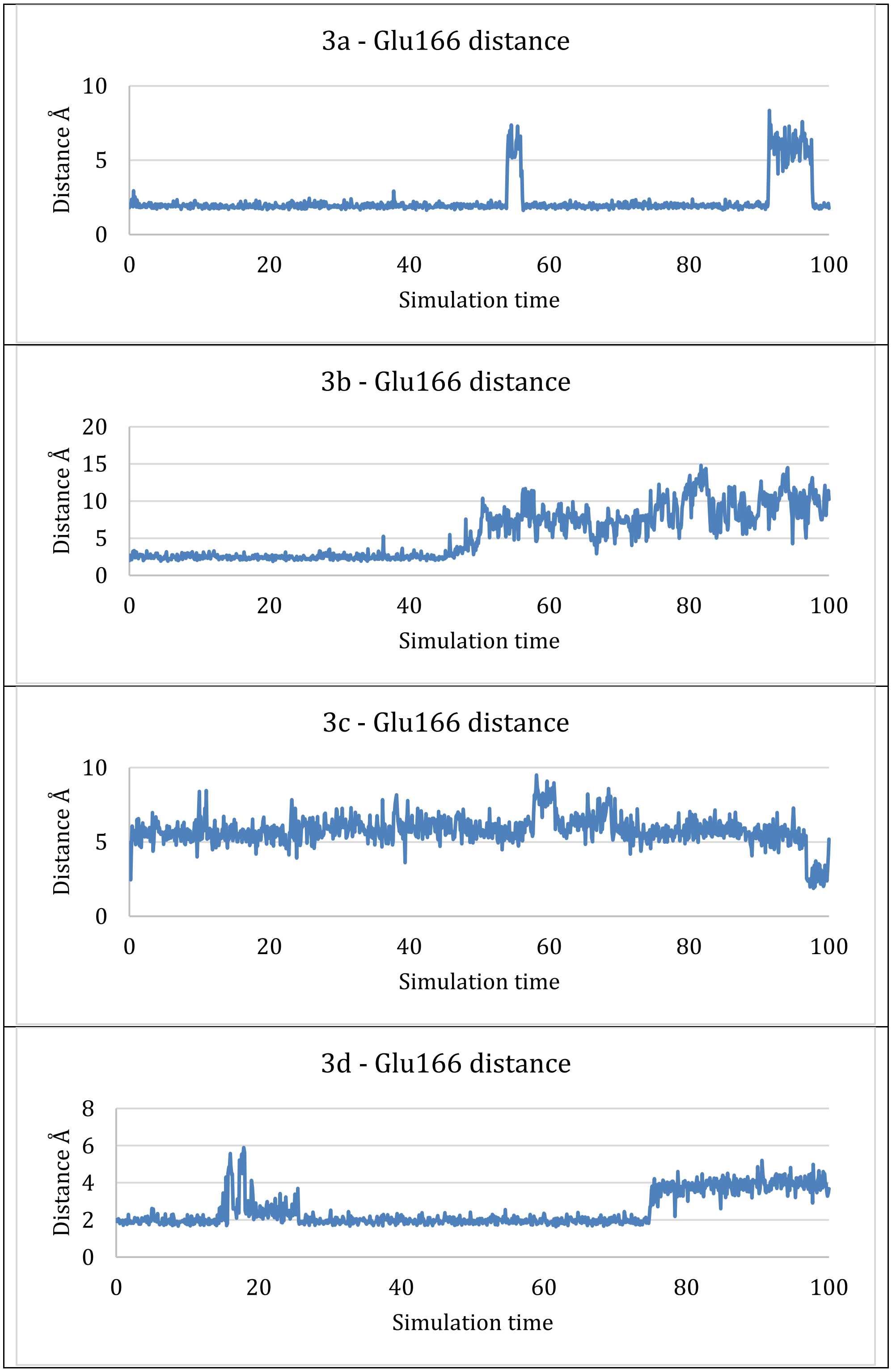

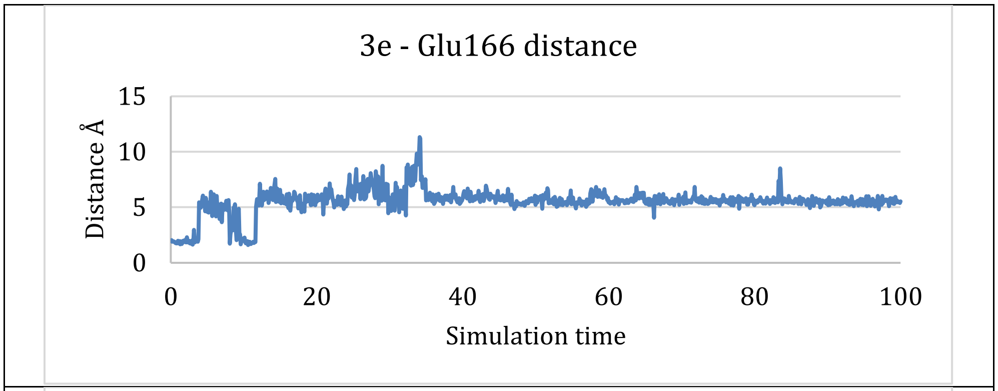
3e - Glu166 distance
Distance Å
15
10
5
0
0
20
40
60
80
100
Simulation time

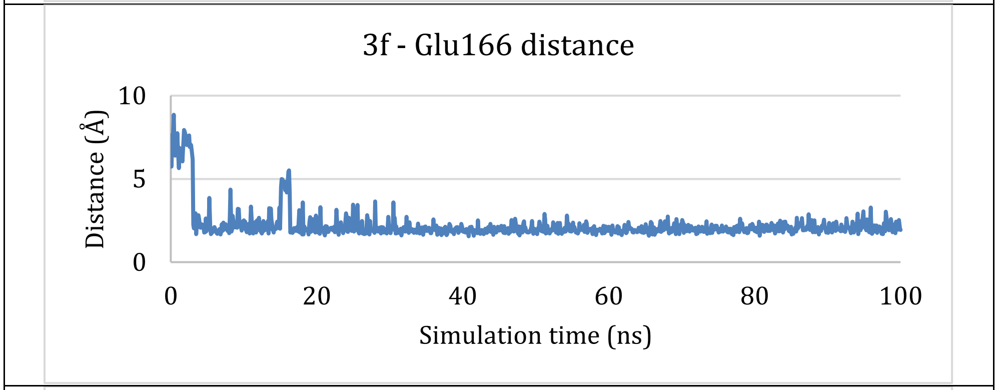
3f - Glu166 distance
Distance (Å)
10
5
0
0
20
40
60
80
100
Simulation time (ns)

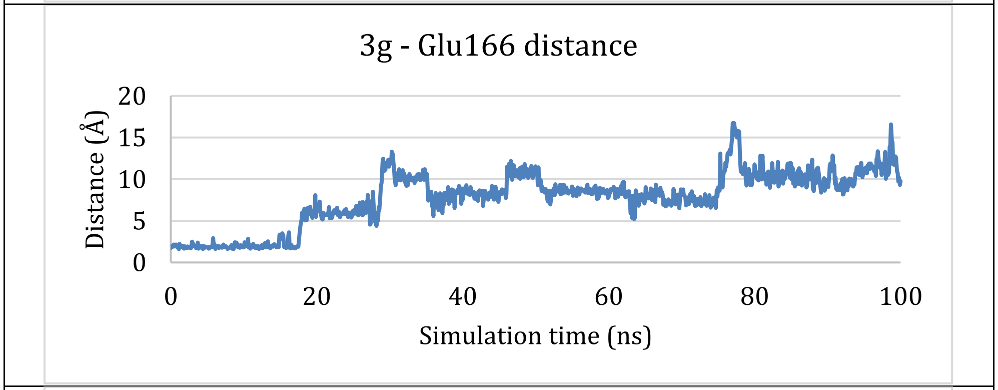
3g - Glu166 distance
Distance (Å)
20
15
10
5
0
0
20
40
60
80
100
Simulation time (ns)

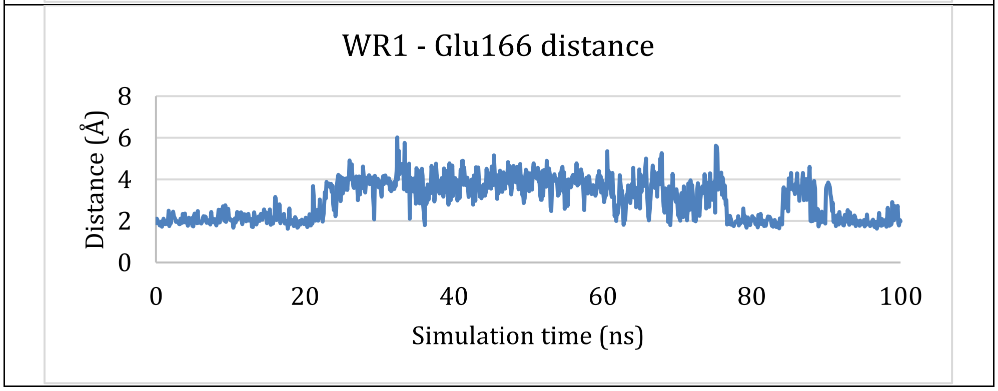
WR1 - Glu166 distance
Distance (Å)
8
6
4
2
0
0
20
40
60
80
100
Simulation time (ns)

Figure SI 4. Histograms and heat maps for SARS-CoV protein-ligand contacts of (**A**) 3d, (**B**) 3e, and (**C**) 3f.

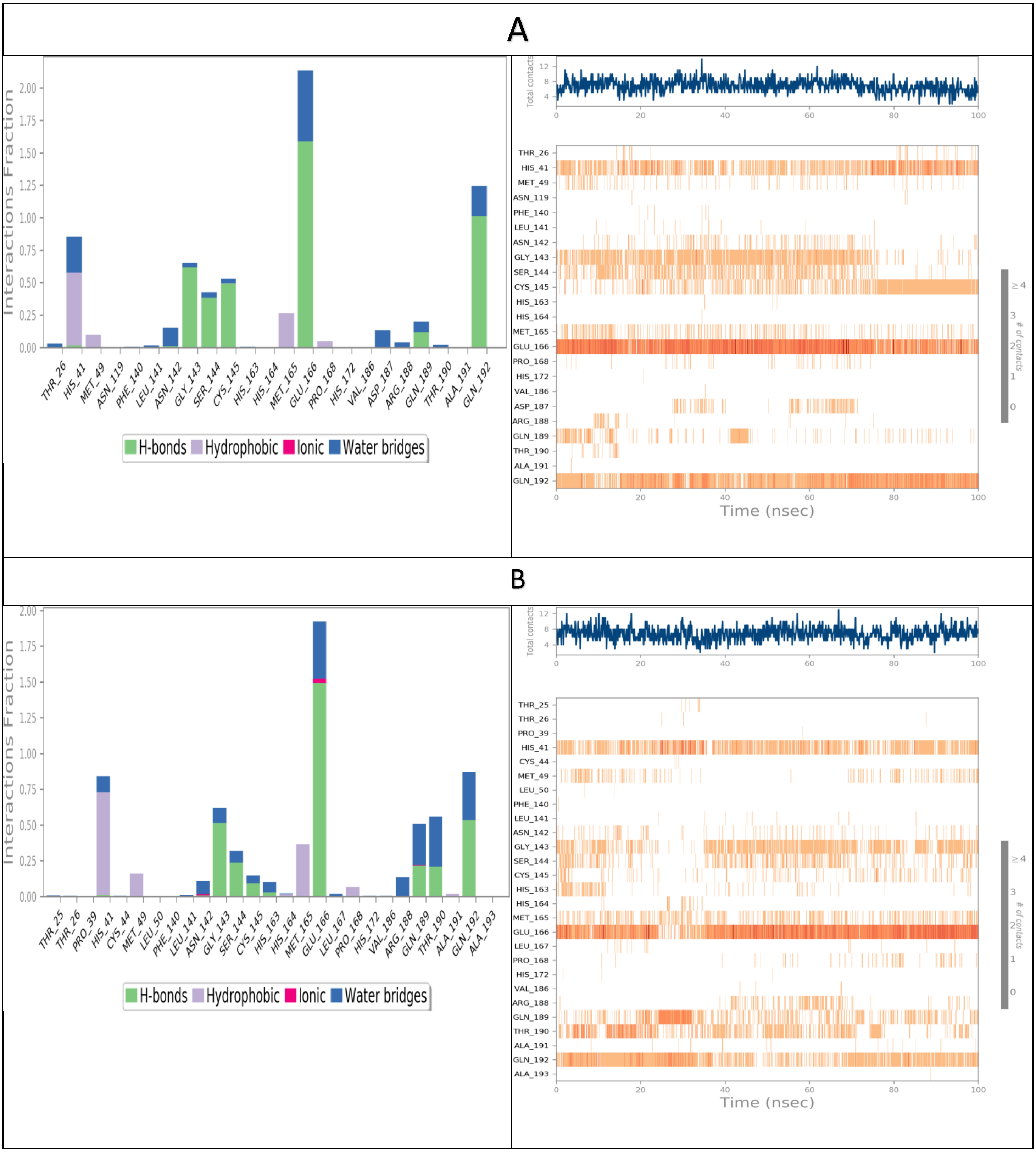

C

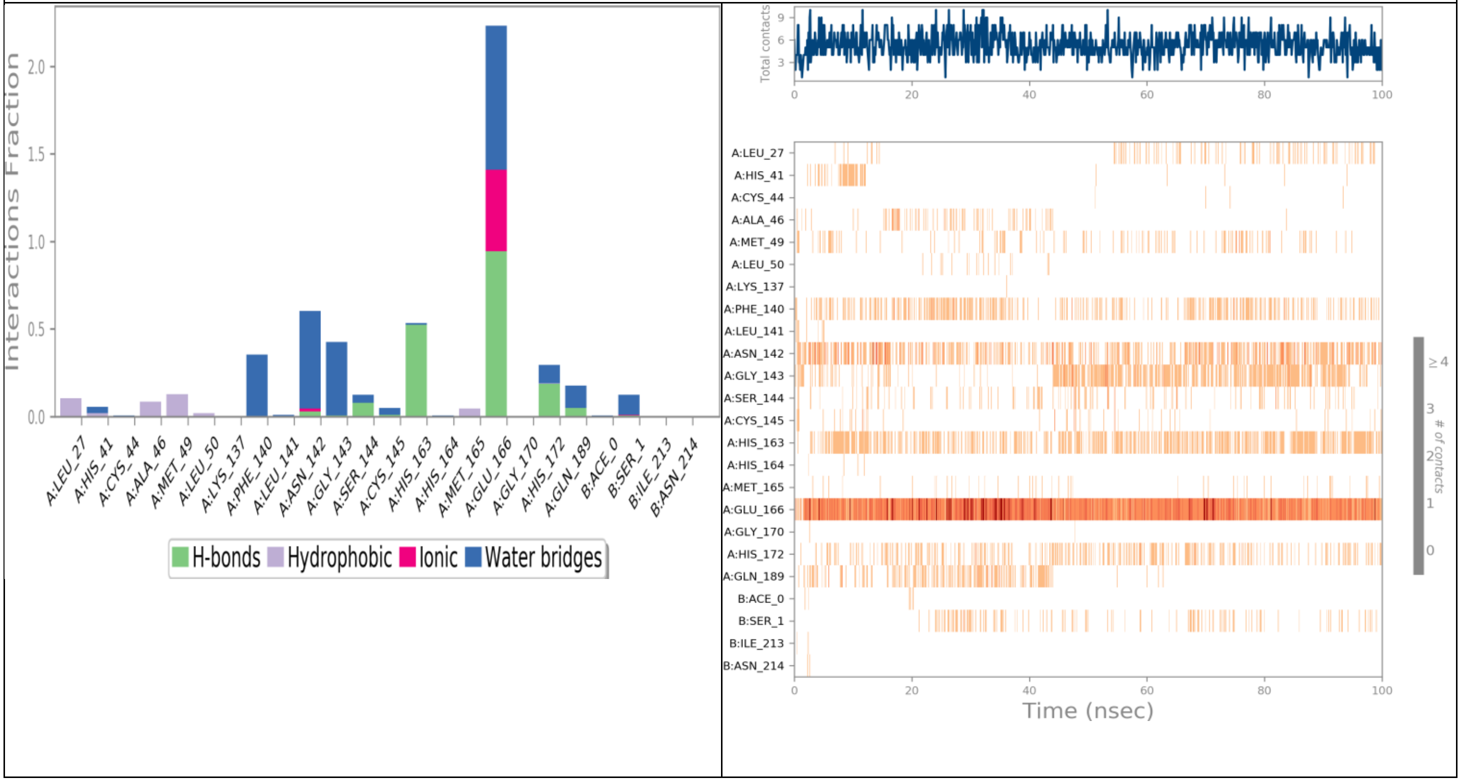

Table SI 1. IC_{50} values of the synthesized compounds (**3a-g**) against SARS-CoV-2 Mpro.

Compound	IC_{50} (μg/mL)
**3a**	4.667
**3b**	5.115
**3c**	11.9
**3d**	88.84
**3e**	16.57
**3f**	22.37
**3g**	51.37

Table SI 2. 2D pictures representing the binding interactions of the investigated compounds (**3a-g**) into SARS-CoV Mpro target receptor with the redocked co-crystallized ligand WR1.

Compound	2D
**3a**	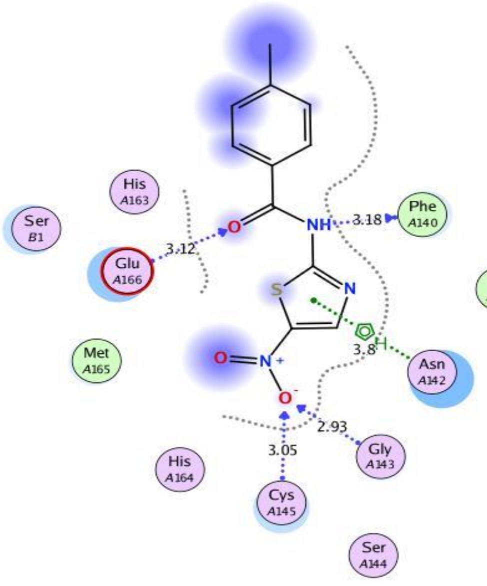
**3b**	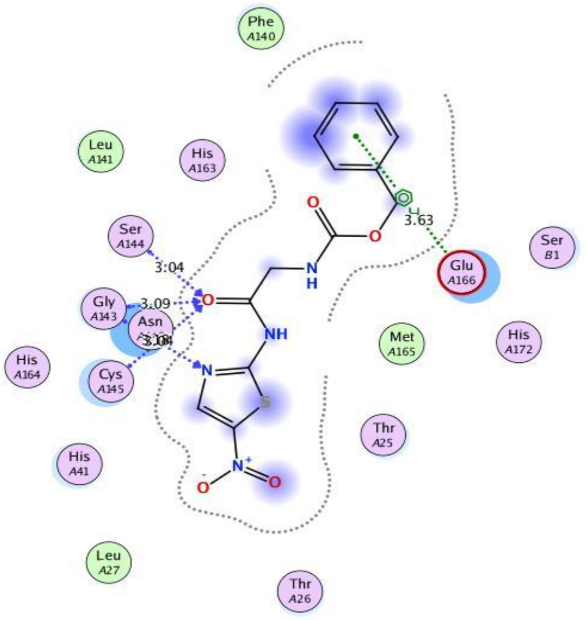

**3c**	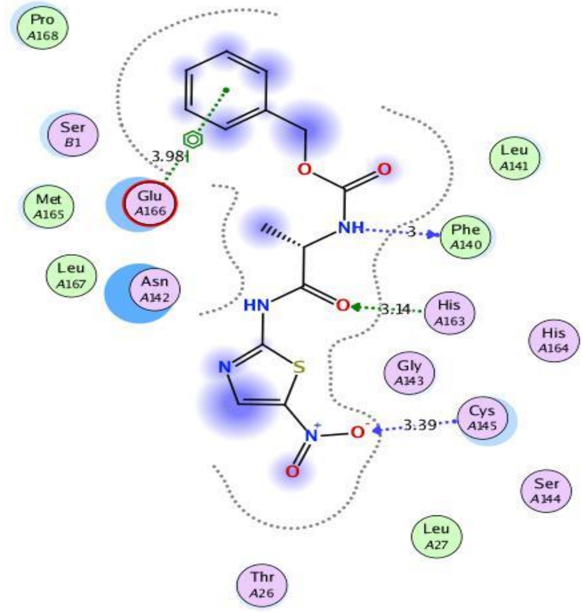Pro A168<br>Ser B1<br>Glu A166<br>Met A165<br>Leu A167<br>Asn A142<br>Leu A141<br>Phe A140<br>His A163<br>His A164<br>Gly A143<br>Cys A145<br>Ser A144<br>Leu A27<br>Thr A26<br>3.98<br>3<br>3.14<br>3.39<br>NH<br>HN<br>O<br>N<br>S
**3d**	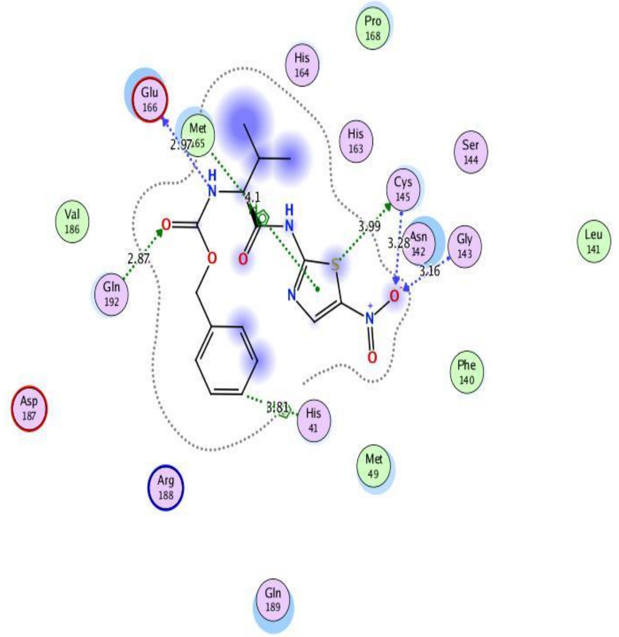Pro 168<br>His 164<br>Glu 166<br>Met 65<br>His 163<br>Ser 144<br>Cys 145<br>Val 186<br>Asn 142<br>Gly 143<br>Leu 141<br>Gln 192<br>Phe 140<br>Asp 187<br>His 41<br>Met 49<br>Arg 188<br>Gln 189<br>2.97<br>4.1<br>3.99<br>3.28<br>3.16<br>2.87<br>3.81

**3e**	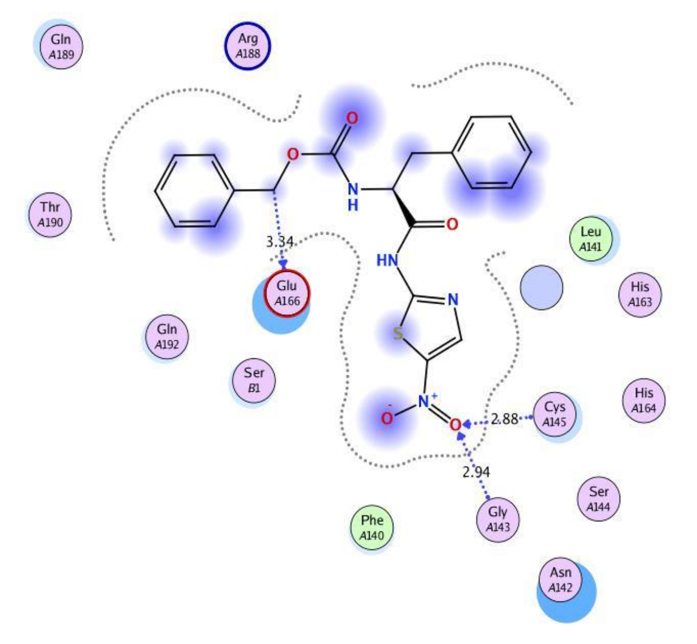Gln A189<br>Arg A188<br>Thr A190<br>3.34<br>Glu A166<br>Gln A192<br>Ser B1<br>Leu A141<br>His A163<br>His A164<br>Cys A145<br>2.88<br>2.94<br>Ser A144<br>Gly A143<br>Phe A140<br>Asn A142
**3f**	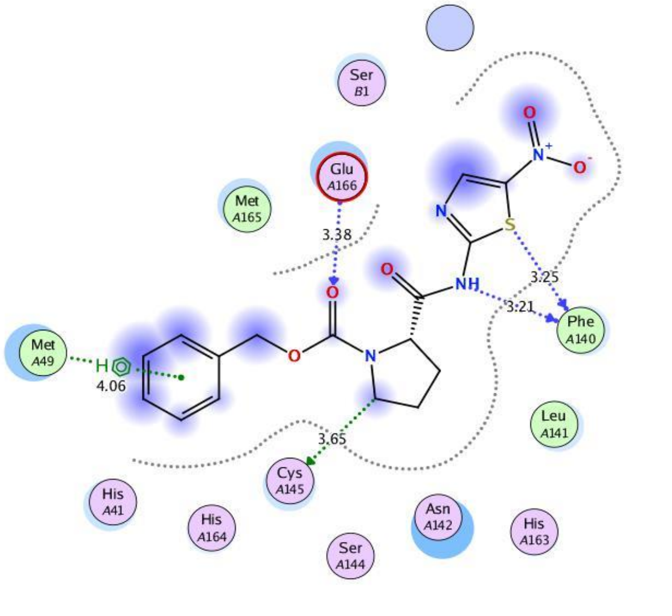Ser B1<br>Glu A166<br>Met A165<br>3.38<br>3.25<br>3.21<br>Phe A140<br>Met A49<br>4.06<br>3.65<br>Leu A141<br>Cys A145<br>His A41<br>His A164<br>Ser A144<br>Asn A142<br>His A163

**3g**	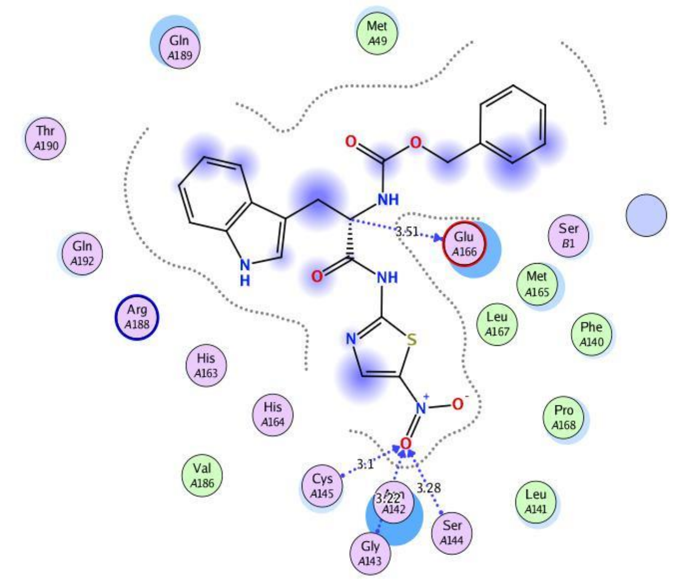Met A49<br>Gln A189<br>Thr A190<br>Gln A192<br>Arg A188<br>His A163<br>His A164<br>Val A186<br>Cys A145<br>Asn A142<br>Gly A143<br>Ser A144<br>Leu A141<br>Pro A168<br>Phe A140<br>Leu A167<br>Met A165<br>Ser B1<br>Glu A166<br>3.51<br>3.1<br>3.22<br>3.28<br>NH<br>H<br>N<br>O<br>S
**WR1**	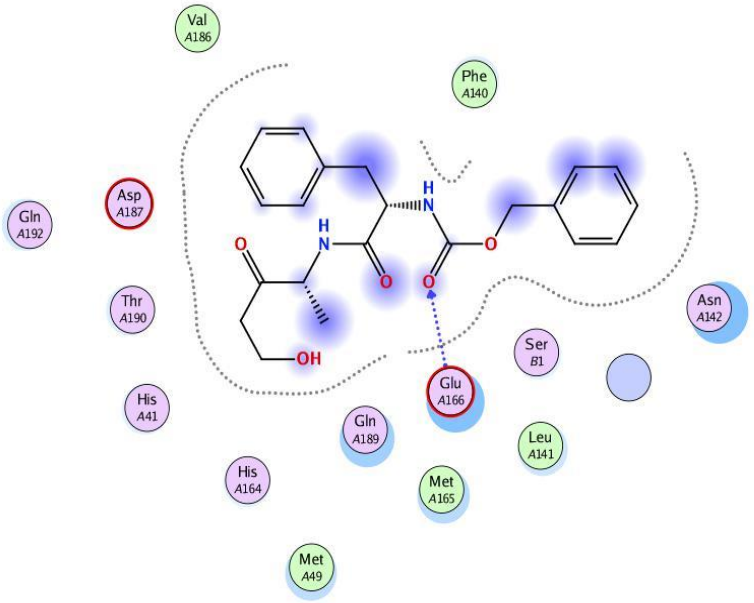Val A186<br>Phe A140<br>Asp A187<br>Gln A192<br>Thr A190<br>His A41<br>His A164<br>Gln A189<br>Met A165<br>Met A49<br>Glu A166<br>Ser B1<br>Leu A141<br>Asn A142<br>H<br>N<br>O<br>OH

SI 1. NMR analysis data of the target compounds (3a-g).

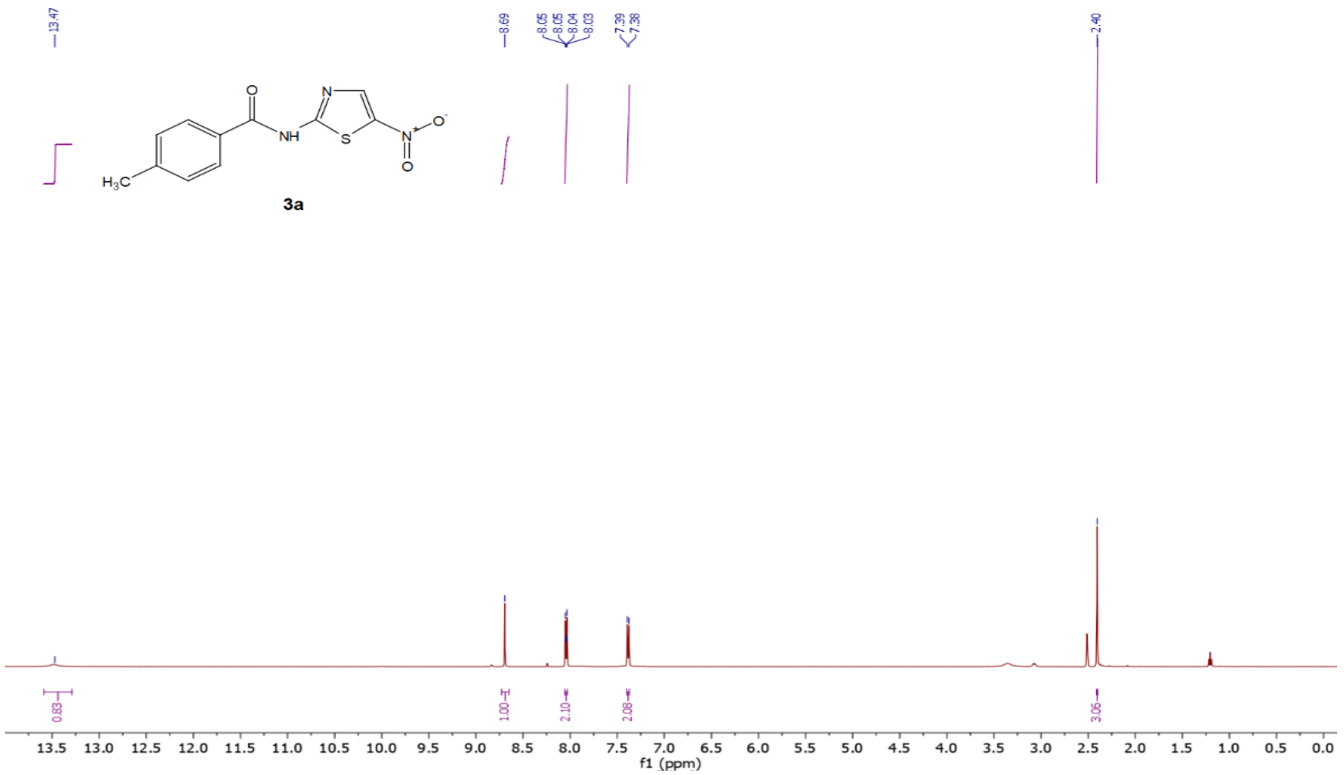

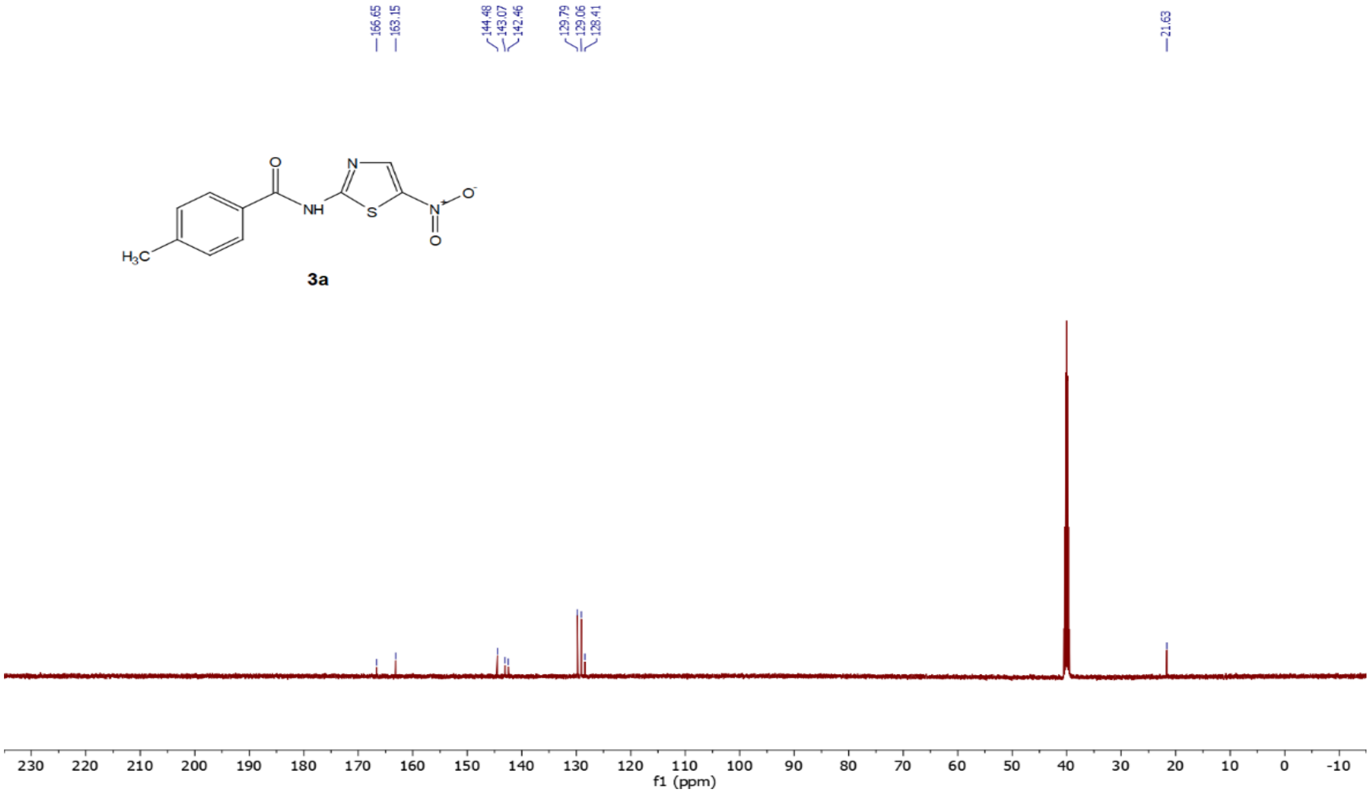

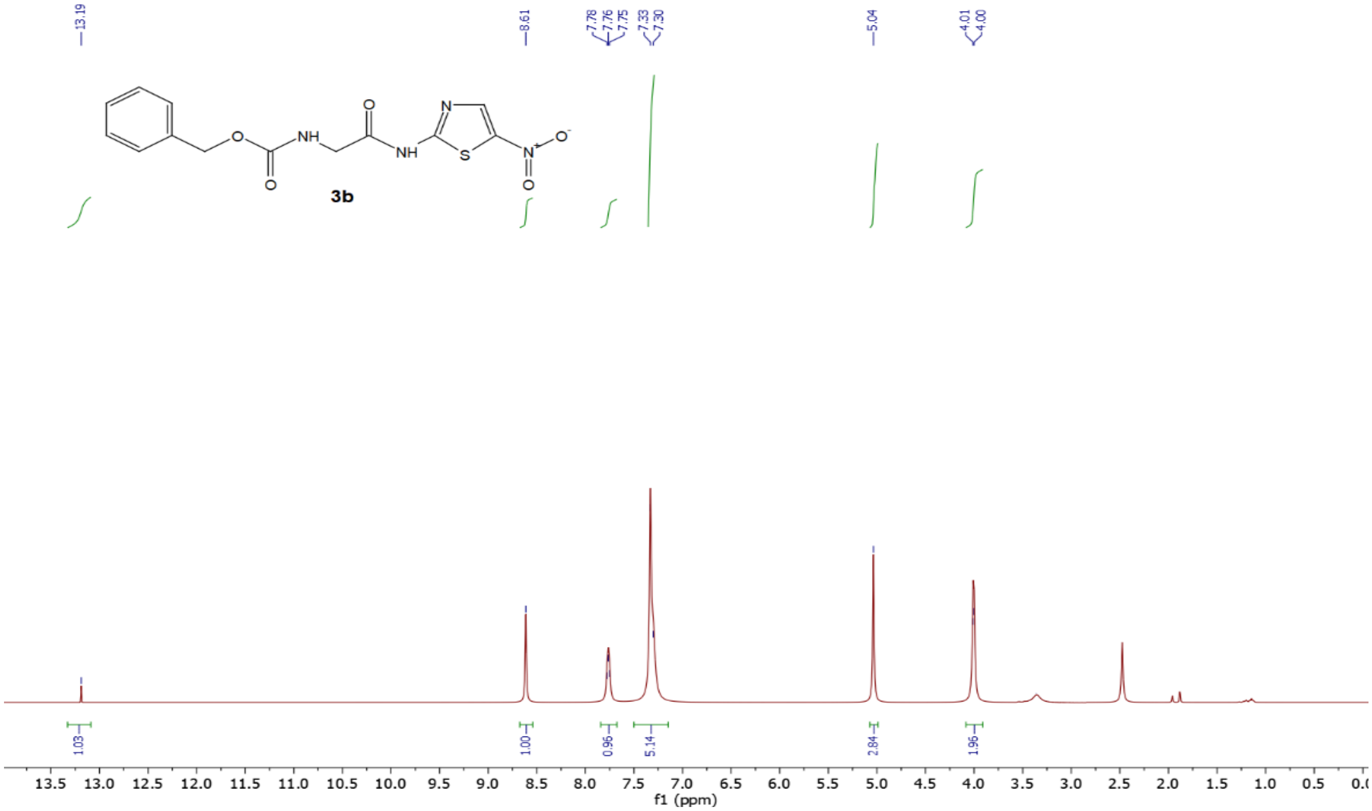
13.19
8.61
7.78
7.76
7.75
7.33
7.30
5.04
4.01
4.00
NH
O
N
S
3b
1.03
1.00
0.96
5.14
2.84
1.96
13.5 13.0 12.5 12.0 11.5 11.0 10.5 10.0 9.5 9.0 8.5 8.0 7.5 7.0 6.5 6.0 5.5 5.0 4.5 4.0 3.5 3.0 2.5 2.0 1.5 1.0 0.5
f1 (ppm)

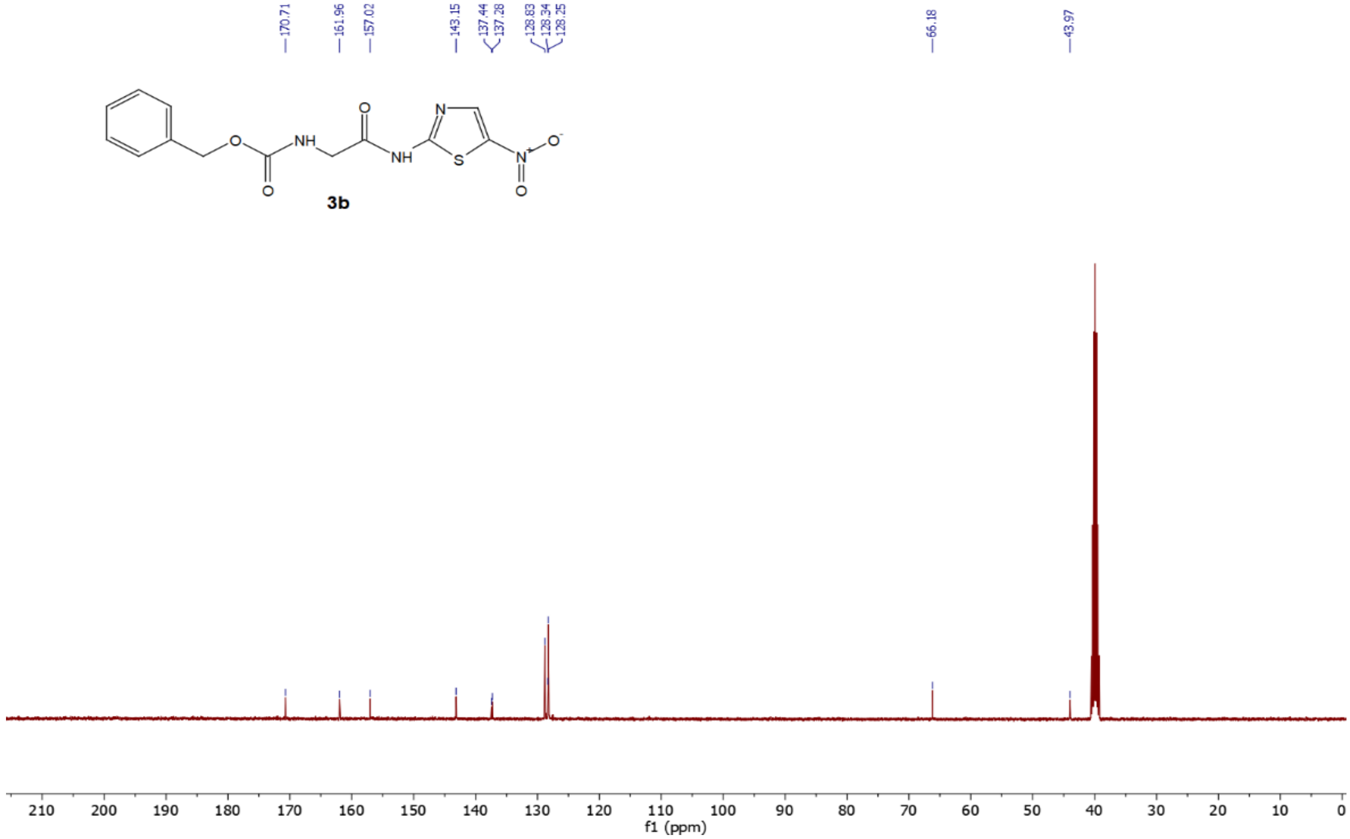
170.71
161.96
157.02
143.15
137.44
137.28
128.83
128.34
128.25
66.18
43.97
NH
O
N
S
3b
210 200 190 180 170 160 150 140 130 120 110 100 90 80 70 60 50 40 30 20 10 0
f1 (ppm)

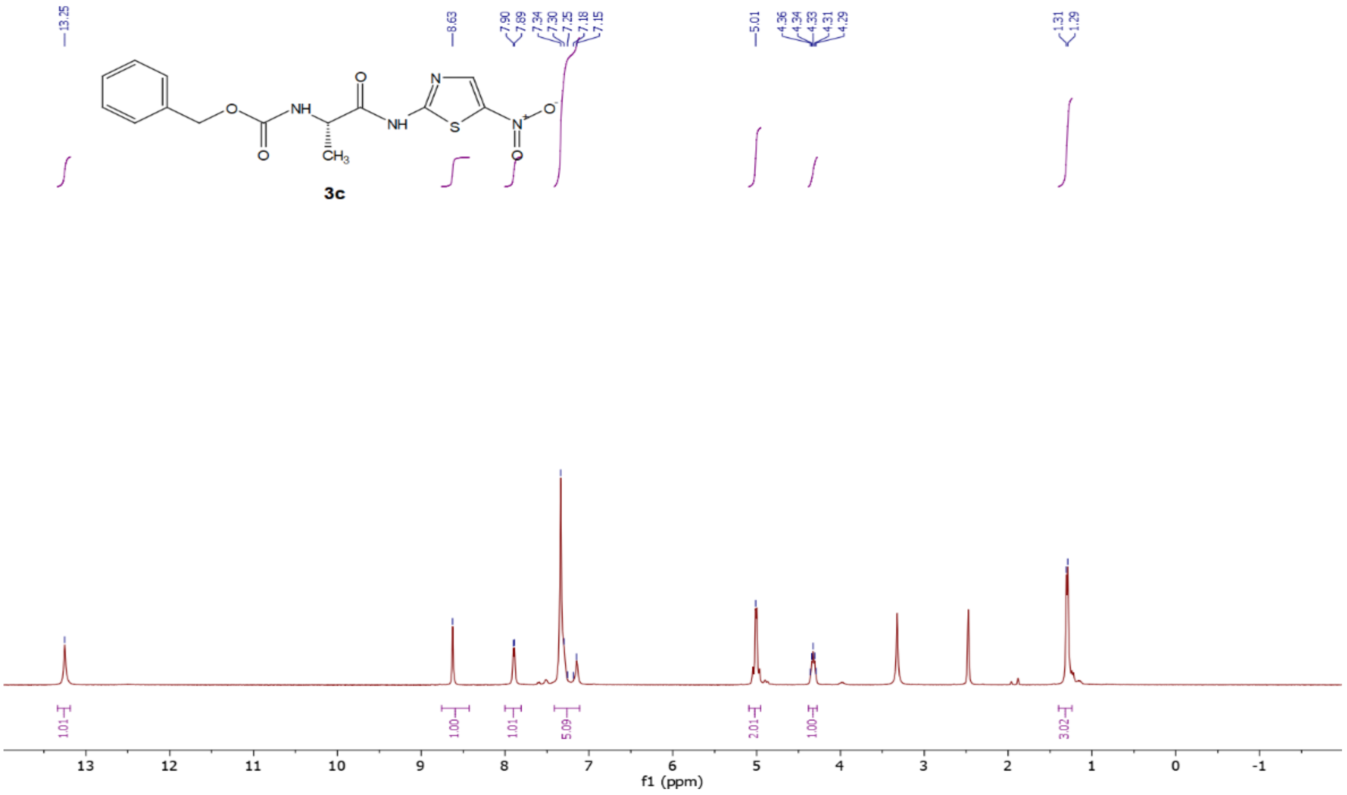
13.25
8.63
7.90
7.89
7.34
7.30
7.25
7.18
7.15
5.01
4.36
4.34
4.33
4.31
4.29
1.31
1.29
3c
1.01
1.00
1.01
5.09
2.01
1.00
3.02
f1 (ppm)

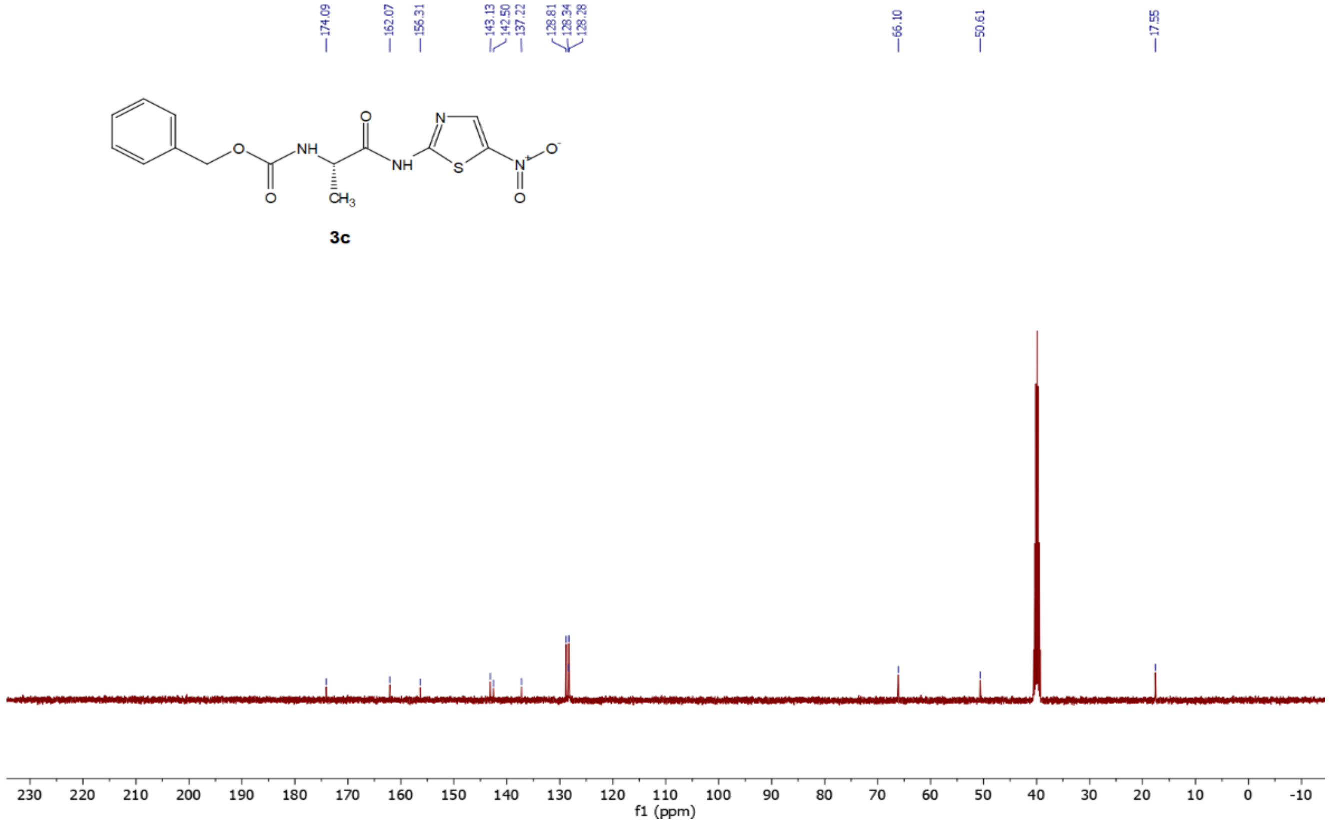
174.09
162.07
156.31
143.13
142.50
137.22
128.81
128.34
128.28
66.10
50.61
17.55
3c
f1 (ppm)

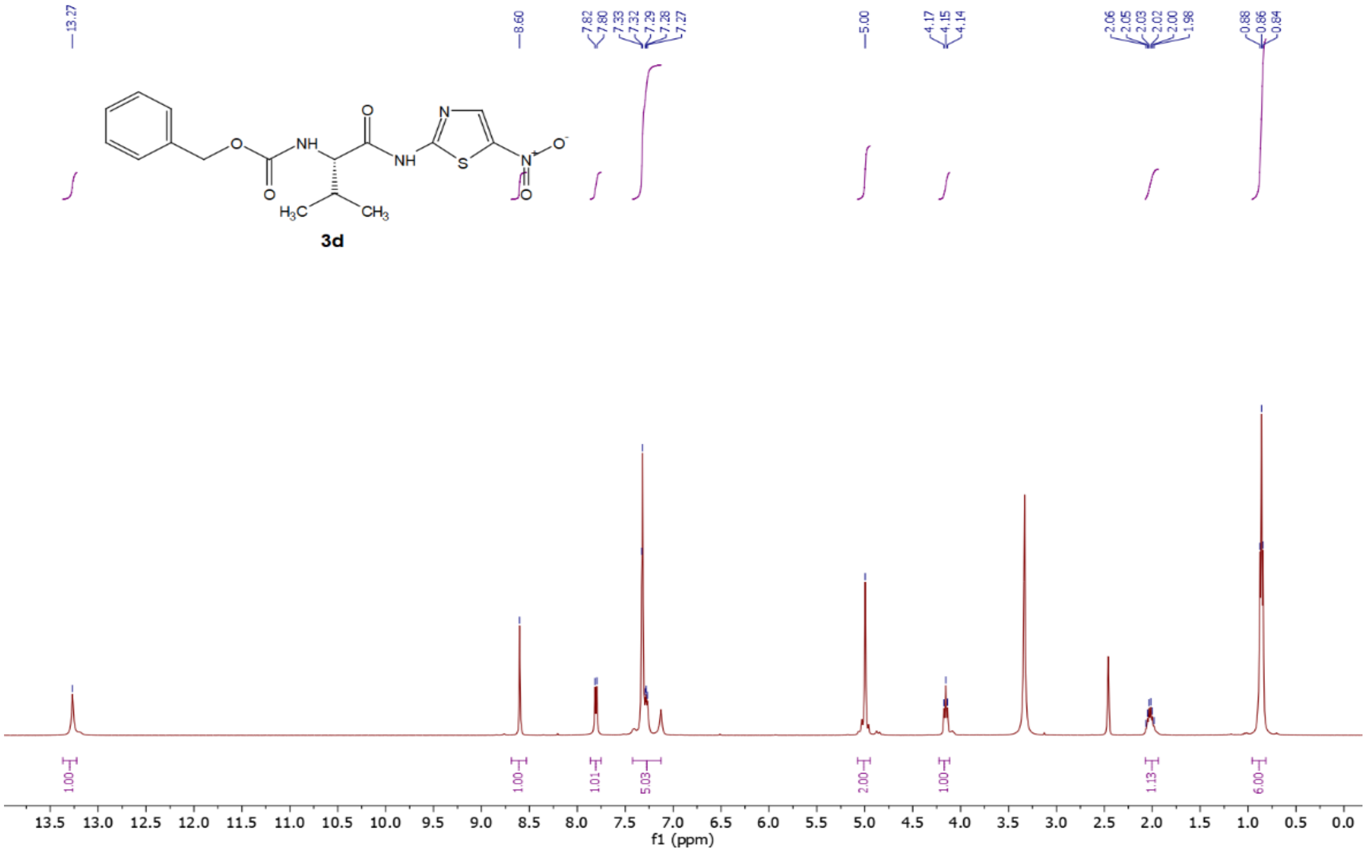
3d
f1 (ppm)

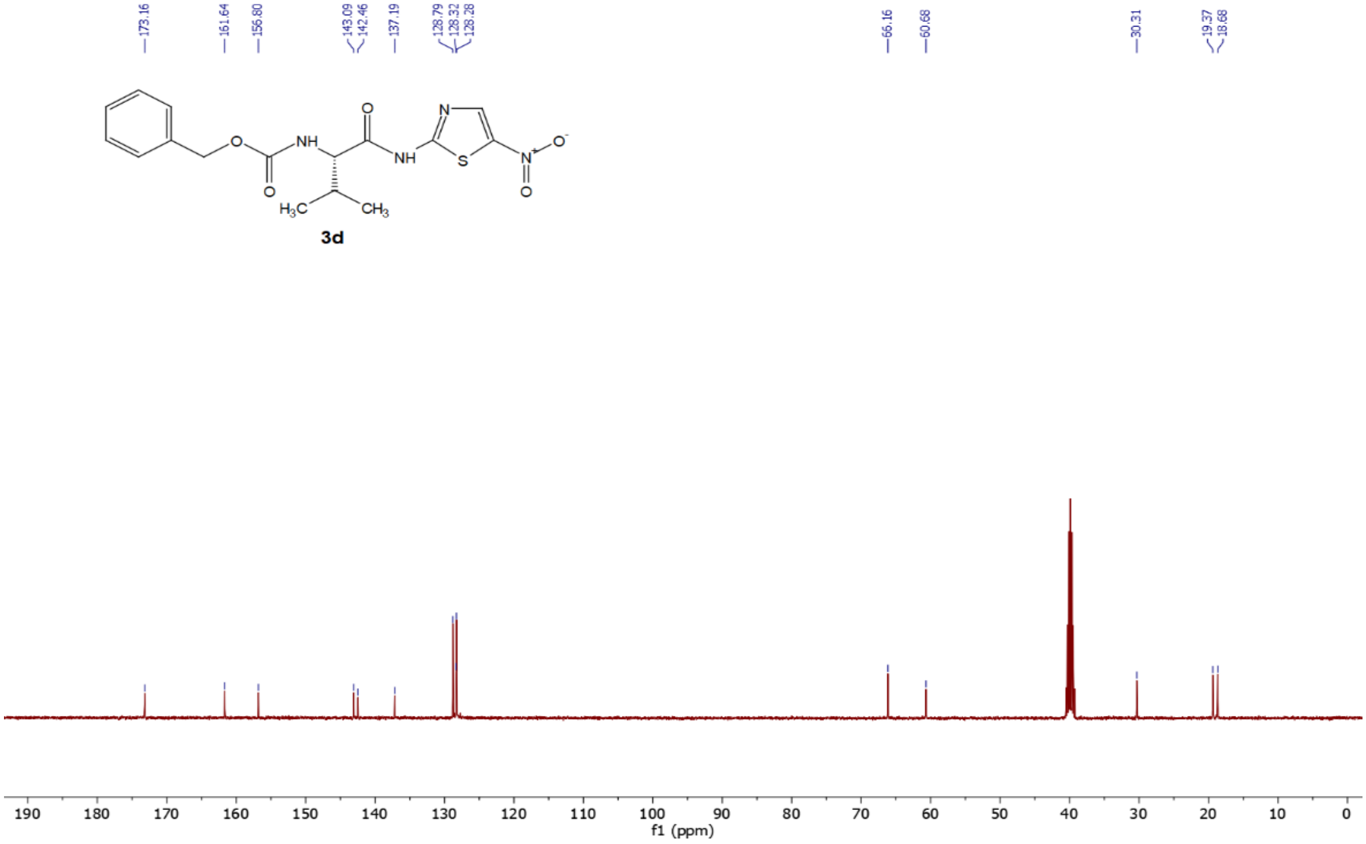
3d
f1 (ppm)

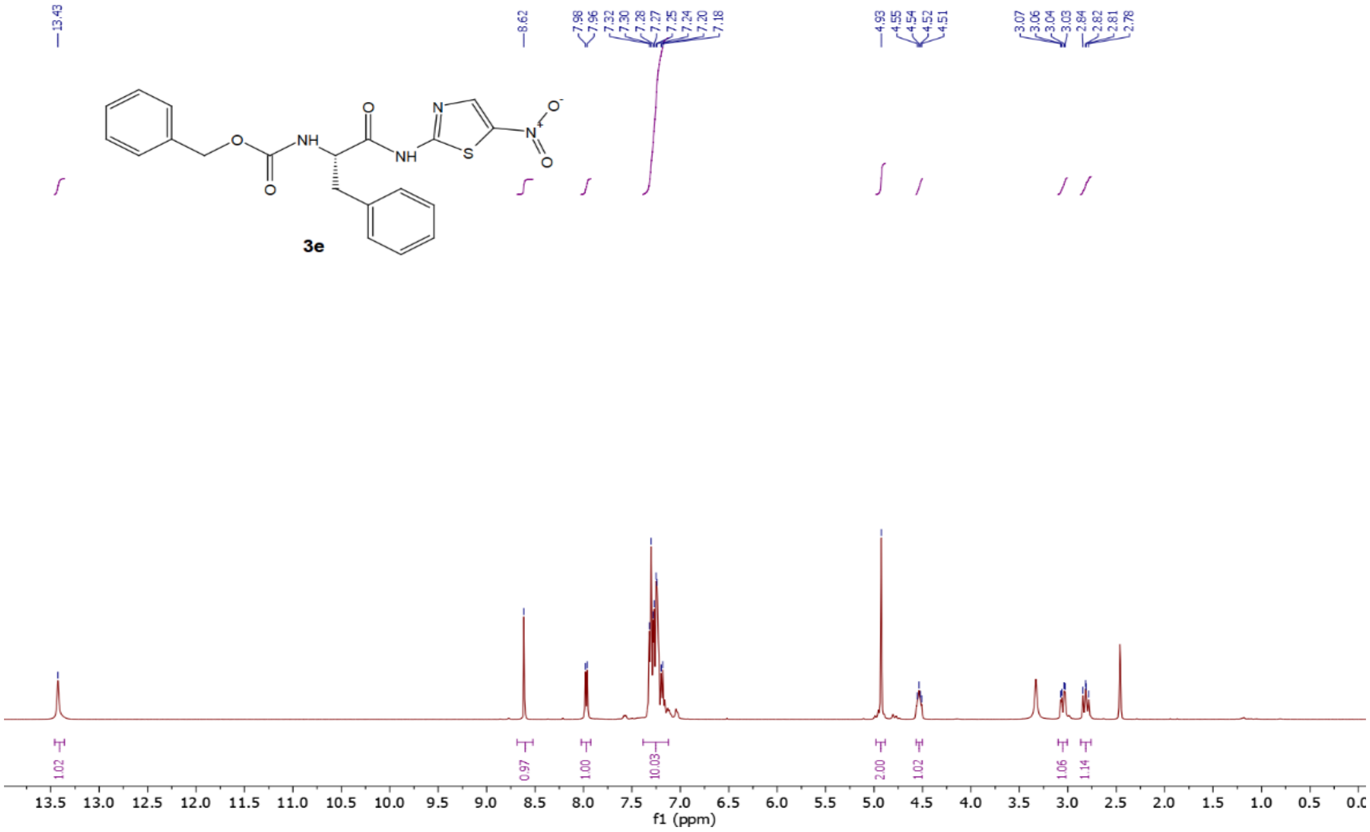
3e
f1 (ppm)

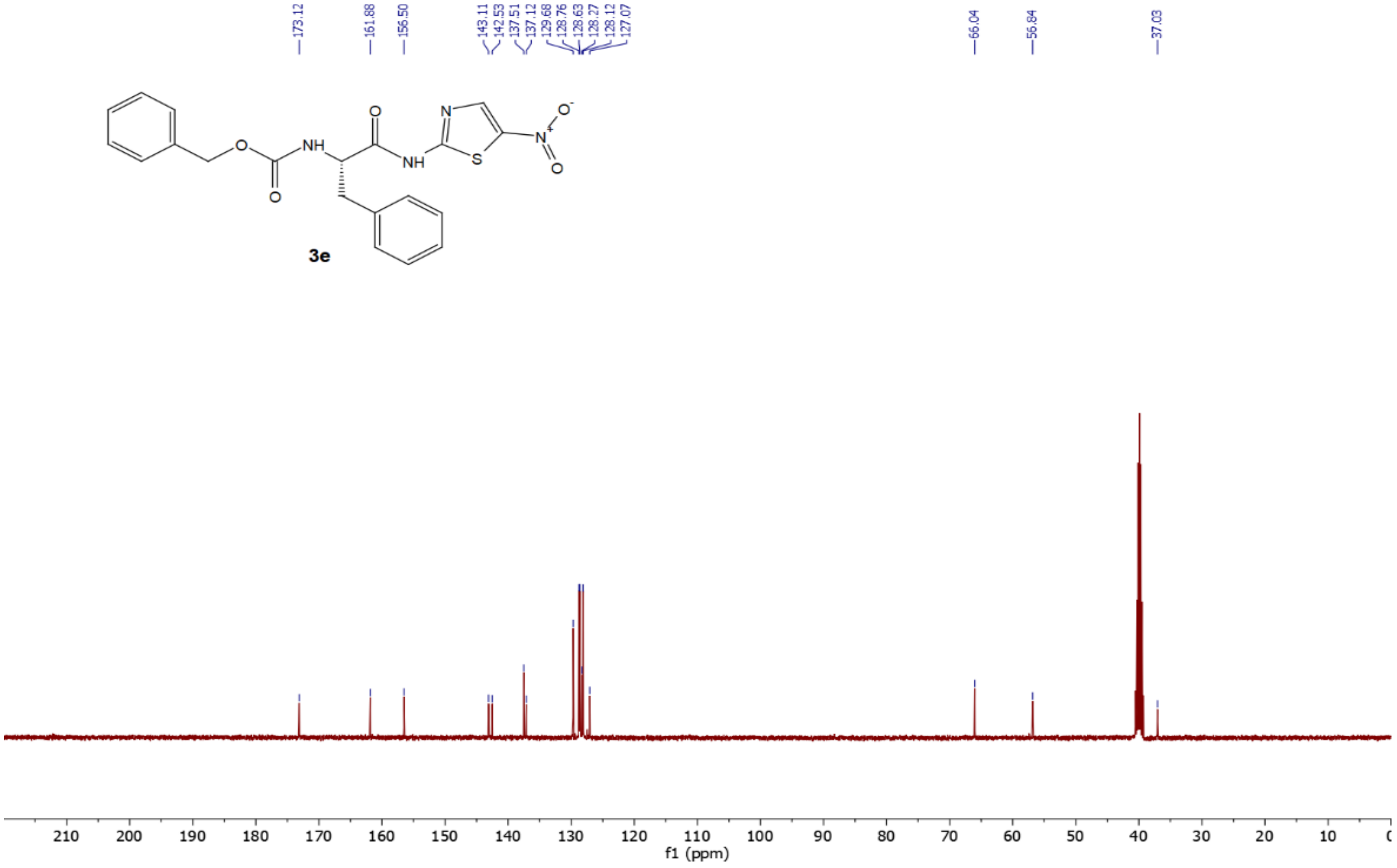
3e
f1 (ppm)

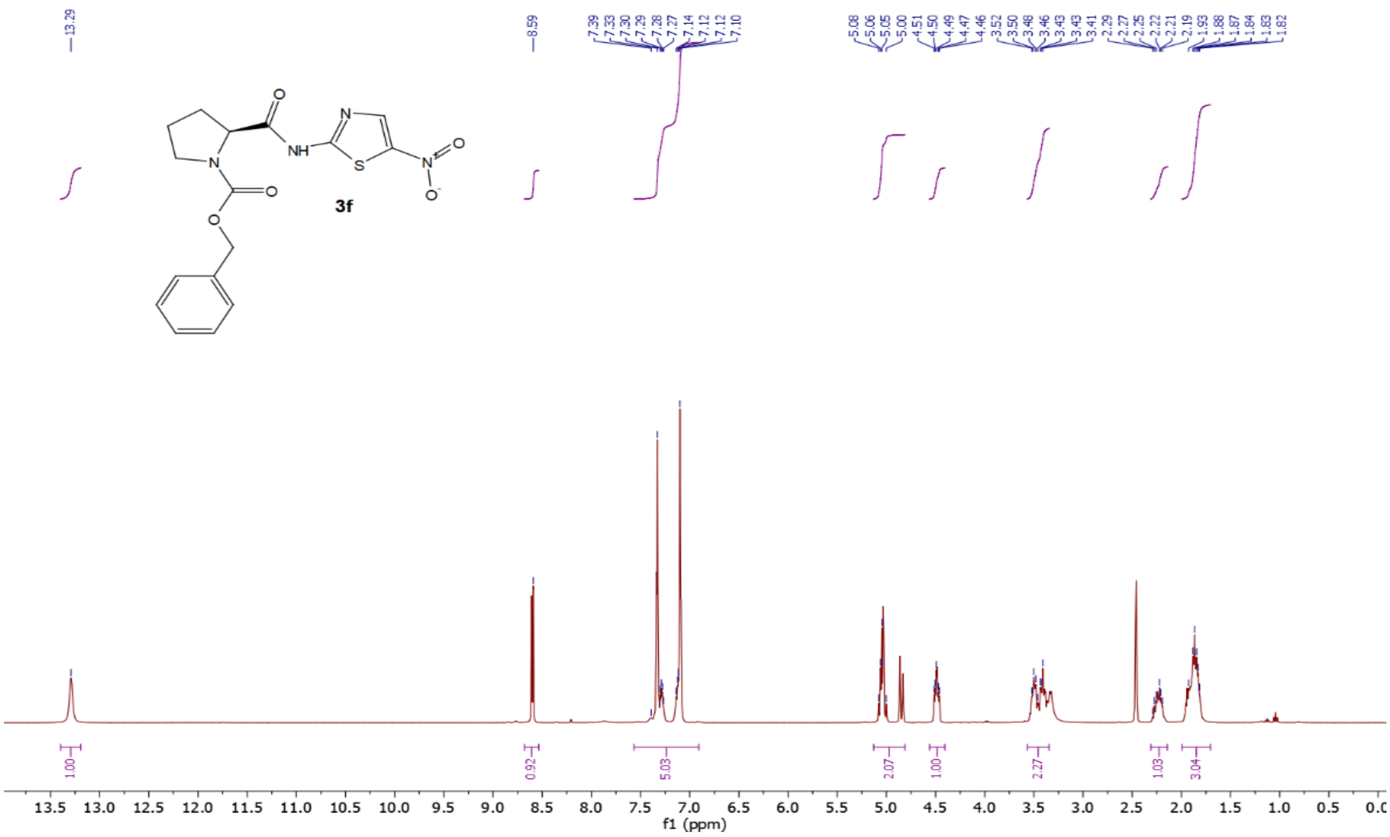
3f
13.29
8.59
7.39 7.33 7.30 7.29 7.28 7.27 7.14 7.12 7.12 7.10
5.08 5.06 5.05 5.00 4.51 4.50 4.49 4.47 4.46 3.52 3.50 3.48 3.46 3.43 3.43 3.41 2.29 2.27 2.25 2.22 2.21 2.19 1.93 1.88 1.87 1.84 1.83 1.82
1.00
0.92
5.03
2.07
1.00
2.27
1.03
3.04
f1 (ppm)

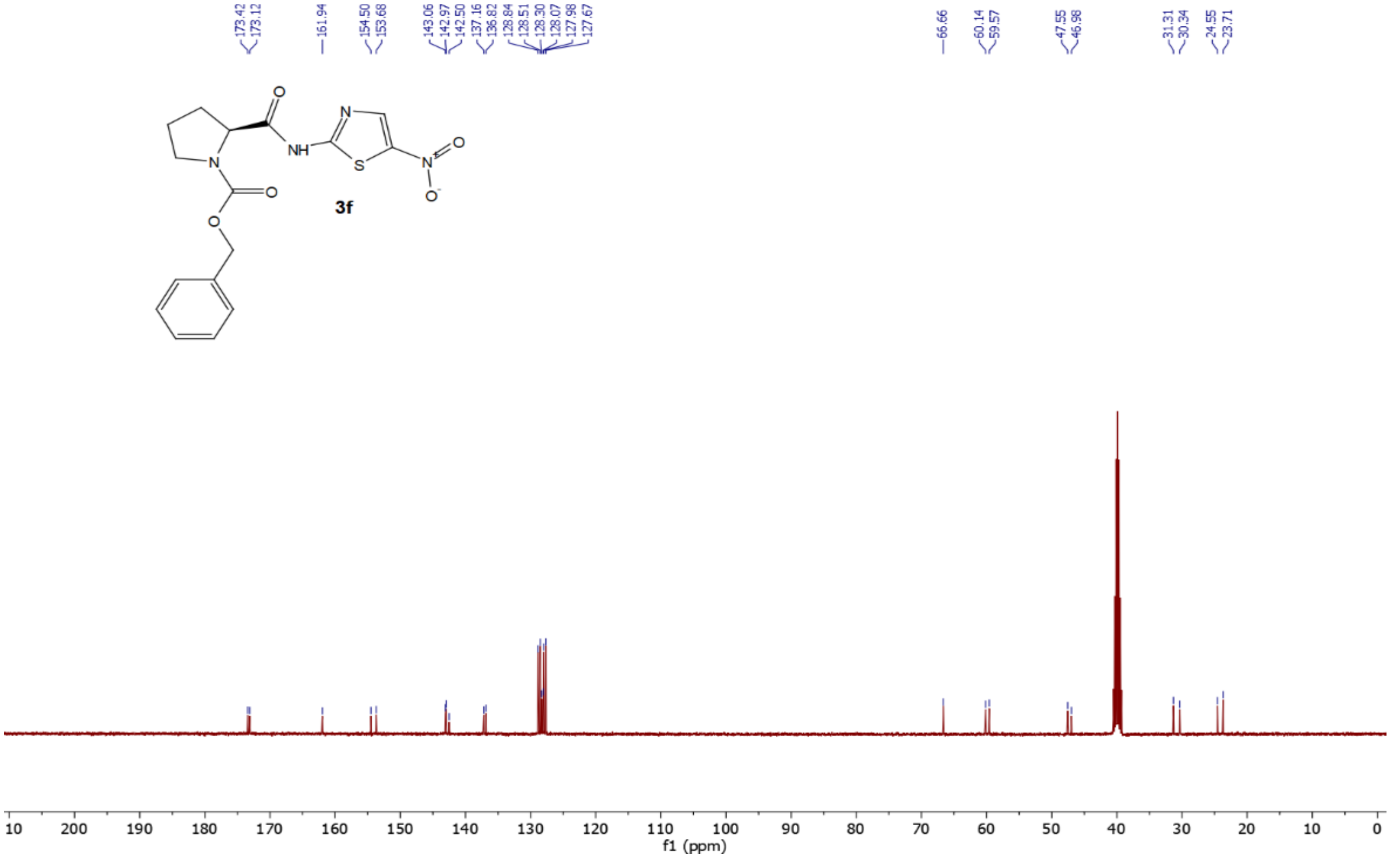
3f
173.42 173.12
161.94
154.50 153.68
143.06 142.97 142.50 137.16 136.82 128.84 128.51 128.30 128.07 127.98 127.67
66.66
60.14 59.57
47.55 46.98
31.31 30.34
24.55 23.71
f1 (ppm)

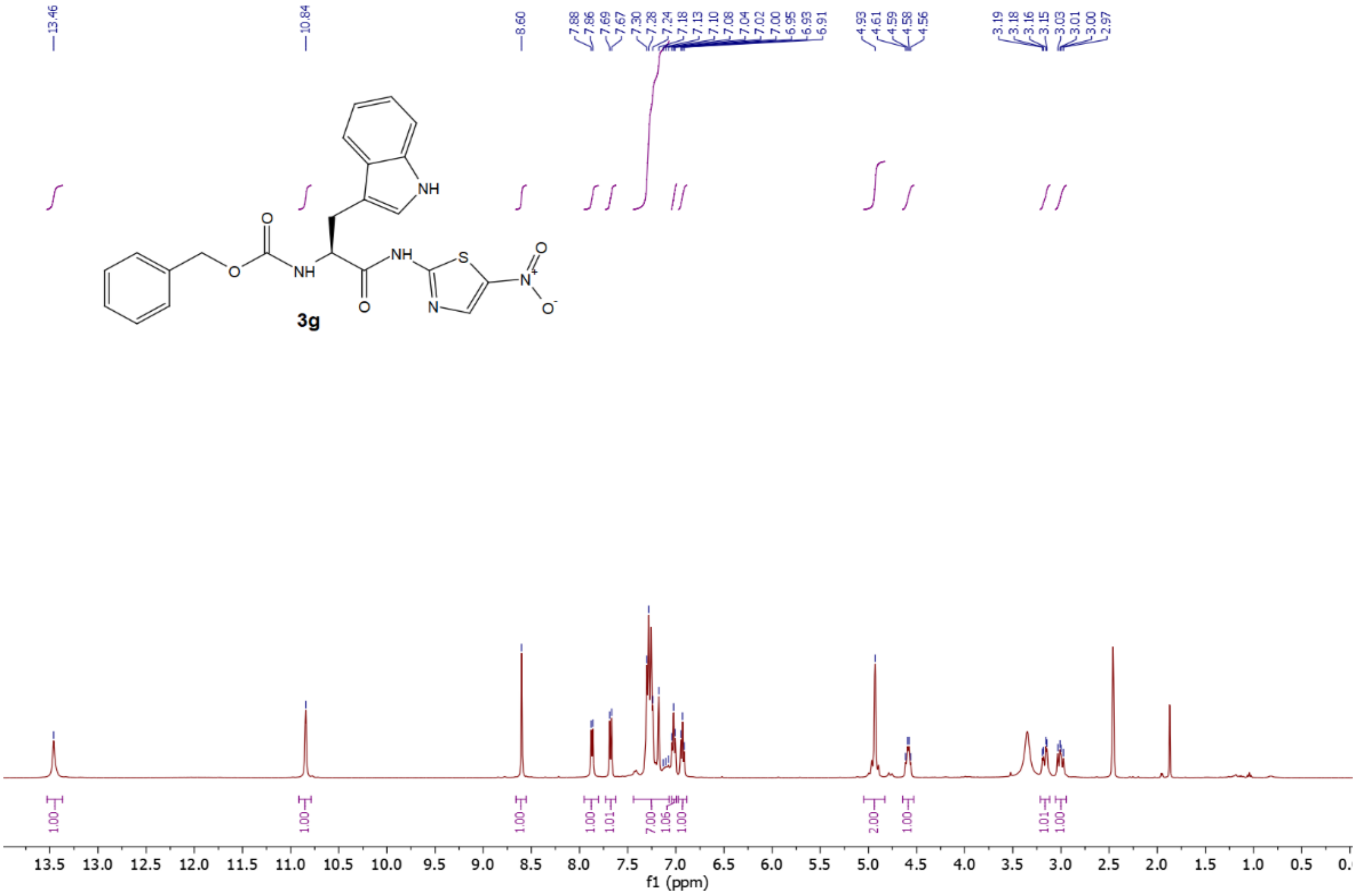
13.46
10.84
8.60
7.88
7.86
7.69
7.67
7.30
7.28
7.24
7.18
7.13
7.10
7.08
7.04
7.02
7.00
6.95
6.93
6.91
4.93
4.61
4.59
4.58
4.56
3.19
3.18
3.16
3.15
3.03
3.01
3.00
2.97
NH
O
NH
O
NH
S
N
N+
O
O-
3g
1.00
1.00
1.00
1.00
1.01
7.00
1.06
1.00
2.00
1.00
1.01
1.00
f1 (ppm)

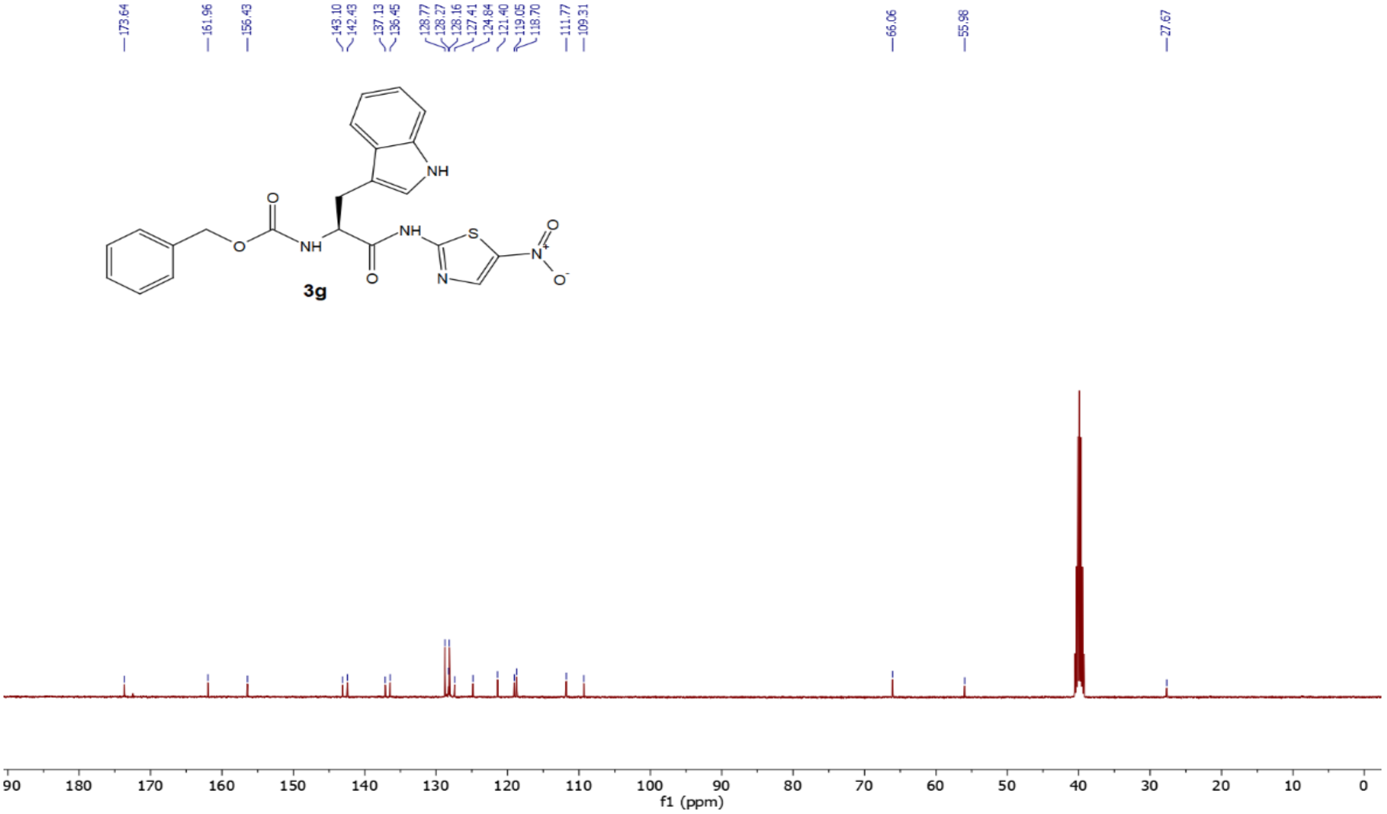
173.64
161.96
156.43
143.10
142.43
137.13
136.45
128.77
128.27
128.16
127.41
124.84
121.40
119.05
118.70
111.77
109.31
66.06
55.98
27.67
NH
O
NH
O
NH
S
N
N+
O
O-
3g
f1 (ppm)

Materials and Methods

SI 2. MTT cytotoxicity assay.

The VERO-E6 cells were kept in 96 well-plates at 37°C in 5% CO_2 for 24 h to be cultivated. The tested compounds were diluted with DMEM in HA plate in triplicates as mentioned before and then poured onto the prepared cells after washing twice by sterile 1x phosphate buffer saline (PBS). 24 h later, the cell monolayers were washed three times with sterile 1x PBS after removal of the supernatant followed by the addition of the MTT solution into each individual well (20 µl of 5 mg/ml stock solution) which was kept at 37°C for 4 h. The formed formazan crystals were dissolved using an acidified isopropanol (200 µl) and the absorbance of their solutions were recorded through a multi-well plate reader (λ_{max} = 540 nm) against a reference wavelength (λ_{max}= 620 nm). Finally, the cytotoxicity % of the tested compounds compared to the control cells (untreated cells) was calculated as follow:

$$\%\ \text{Cytotoxicity} = \frac{(\text{the absorbance of untreated cells } - \text{absorbance of treated cells})\ \text{X}\ 100}{\text{the absorbance of treated cells}}$$

SI 3. Inhibitory concentration 50 (IC_{50}) determination.

The Vero-E6 cells (2.4×104) were kept overnight at 37°C in 5% CO_2 inside 96-well tissue culture plates. 1x PBS solution was used to wash the cell monolayers for only one time which were then treated with different serial dilutions of the examined compounds together with a fixed dilution from the virus (hCoV-19/Egypt/NRC-03/2020 (Accession Number on GSAID: EPI_ISL_430820)) following $TCID_{50}$ test and kept at RT for 1 h before starting incubation. Also, the cell monolayers were subjected to DMEM (100 µl) with different concentrations of the test samples and virus and left at 37°C for 72 h in a 5% CO_2. Then, 4% paraformaldehyde (100 µl) was used for cell fixation (2 h) followed by the staining step with 0.1% crystal violet in distilled H_2O (50 µl) at RT for 15 min. Absolute CH_3OH (100 µl) was added to dissolve the crystal violet dye per well to measure the optical density of the produced color using Anthos Zenyth 200rt plate reader at 570 nm.[1] The IC_{50} value for each tested compound which is corresponding to its minimum concentration required to reduce the virus infectivity by 50% in comparison to the virus control was calculated.

SI 4. Methodology and protocol of 3CL Protease (SARS-CoV-2) assay.

The *3CL Protease Assay Kit* is designed to measure 3CL Protease activity for screening and profiling applications, in a homogeneous assay with no time-consuming washing steps. The kit comes in a convenient 96-well format, with purified 3CL Protease, fluorogenic substrate, and 3CL Protease assay buffer for 100 enzyme reactions. 3CL inhibitor GC376 is also included as a positive control.

Protocol

Add **0.5 M DTT** to **3CL Protease Assay Buffer** so final DTT concentration is 1 mM. For example, add 10 µl of **0.5 M DTT** to 5 ml assay buffer. (DTT should be added just before use. Prepare only enough DTT-containing buffer as required for the assay. Store the remaining assay buffer at -20°C).

2) Thaw **3CL Protease** on ice. Upon first thaw, briefly spin tube containing enzyme to recover the full content of the tube. Aliquot **3CL Protease** into single use aliquots. Store remaining undiluted enzyme in aliquots at -80°C. Note: **3CL Protease** enzyme is sensitive to freeze/thaw cycles. Do not re-use dilute enzyme.

3) Dilute **3CL Protease** in **Assay buffer** (with 1 mM DTT) at 3-5 ng/µl (90-150 ng per reaction).

4) Add 30 µl **diluted 3CL Protease** enzyme solution to wells designated as “Positive Control”, “Inhibitor Control” and “Test Sample”. Add 30 µl **Assay buffer** (with 1 mM DTT) to the “Blank” wells.

**Component**	**Positive Control**	**Test Sample**	**Inhibitor Control**	**Blank**
3CL Protease (3-5 ng/µl)	30 µl	30 µl	30 µl	–
Assay Buffer (with DTT)	–	–	–	30 µl
GC376 (500 µM)	–	–	10 µl	–
Test Inhibitor	–	10 µl	–	–
Inhibitor Buffer (no inhibitor)	10 µl	–	–	10 µl
Substrate solution	10 µl	10 µl	10 µl	10 µl
**Total**	**50 µl**		**50 µl**	**50 µl**

5) Dilute 50 µg **GC376** in 200 µl water to obtain a 500 µM solution. Aliquot and store remaining solution in aliquots at -80°C. Add 10 µl **GC376** (500 µM) to the wells labeled “Inhibitor Control”.

6) Prepare the inhibitor solution.

The final concentration of DMSO in the assay should not exceed 1%. If the inhibitor compound is dissolved in DMSO, make a 100-fold higher concentration of the compound than the highest

concentration you want to test in DMSO. Then make a 20-fold dilution in 1X assay buffer (at this step the compound concentration is 5-fold higher than the final concentration).

If the inhibitor compound is dissolved in water, make a solution of the compound 5-fold higher than the final concentration in 3CL Protease assay buffer (with 1 mM DTT). For example, diluting 50 µg GC376 in 200 µl water (step 5) creates a 500 µM solution. Adding 10 ul to the assay (final volume 50 µl) results in a 100 µM final concentration.

7) Add 10 µl inhibitor to each well designated "Test Sample". Add 10 µl 1X assay buffer or 5% DMSO (depending on which inhibitor solution is used) to "Blank" and "Positive Control" wells.

8) Preincubate enzyme with the inhibitor for 30 min at room temperature with slow shaking.

9) Dilute 5 mM **3CL Protease substrate** 1:20 in assay buffer with DTT, to make a 250 µM solution. Dilute only enough as is required for the assay.

10) Start reaction by adding 10 µl of the substrate solution to each well (Final concentration of the **3CL Protease substrate** in a 50 µl reaction is 50 µM).

11) Incubate at room temperature for overnight. Seal the plate with the plate sealer. Measure the fluorescence intensity in a microtiter plate-reading fluorimeter capable of excitation at a wavelength 360 nm and detection of emission at a wavelength 460 nm. The fluorescence intensity can also be measured kinetically. "Blank" value is subtracted from all other values.

SI 5. Molecular dynamics (MD) simulations.

The MD simulations were carried out using Desmond simulation package of Schrödinger LLC.[2] The NPT ensemble with the temperature 300 K and a pressure 1 bar was applied in all runs. The simulation length was 100 ns with a relaxation time 1 ps for the ligands. The OPLS3 force field parameters were used in all simulations.[3] The cutoff radius in Coulomb interactions was 9.0 Å. The orthorhombic periodic box boundaries were set 10 Å away from the protein atoms. The water molecules were explicitly described using the transferable intermolecular potential with three points (TIP3P) model.[4, 5] Salt concentration set to 0.15 M NaCl and was built using the System Builder utility of Desmond.[6] The Martyna−Tuckerman−Klein chain coupling scheme with a coupling constant of 2.0 ps was used for the pressure control and the Nosé−Hoover chain coupling scheme for the temperature control.[7, 8] Nonbonded forces were calculated using a RESPA integrator where the short-range forces were updated every step and the long-range forces were updated every three steps. The trajectories were

saved at 20 ns intervals for analysis. The behavior and interactions between the ligands and protein were analyzed using the Simulation Interaction Diagram tool implemented in Desmond MD package. The stability of MD simulations was monitored by looking on the RMSD of the ligand and protein atom positions in time.

SI 6. MM-GBSA calculations.

Simulation interactions diagram panel of Maestro software was used to monitoring interactions contribution in the ligand-protein stability. The molecular mechanics generalized born/solvent accessibility (MM – GBSA) was performed to calculate the ligand binding free energies and ligand strain energies for docked compounds over the last 25 ns with thermal_mmgbsa.py python script provided by Schrodinger which takes a Desmond trajectory file, splits it into individual snapshots, runs the MM-GBSA calculations on each frame, and outputs the average computed binding energy.

References

1. Marques, N. P.; Lopes, C. S.; Marques, N. C. T.; Cosme-Silva, L.; Oliveira, T. M.; Duque, C.; Sakai, V. T.; Hanemann, J. A. C., A preliminary comparison between the effects of red and infrared laser irradiation on viability and proliferation of SHED. *Lasers in medical science* **2019,** *34* (3), 465-471.
2. Release, S., 3: Desmond molecular dynamics system, DE Shaw research, New York, NY, 2017. *Maestro-Desmond Interoperability Tools, Schrödinger, New York, NY* **2017**.
3. Harder, E.; Damm, W.; Maple, J.; Wu, C.; Reboul, M.; Xiang, J. Y.; Wang, L.; Lupyan, D.; Dahlgren, M. K.; Knight, J. L., OPLS3: a force field providing broad coverage of drug-like small molecules and proteins. *Journal of chemical theory and computation* **2016,** *12* (1), 281-296.
4. Jorgensen, W. L.; Chandrasekhar, J.; Madura, J. D.; Impey, R. W.; Klein, M. L., Comparison of simple potential functions for simulating liquid water. *The Journal of chemical physics* **1983,** *79* (2), 926-935.
5. Neria, E.; Fischer, S.; Karplus, M., Simulation of activation free energies in molecular systems. *The Journal of chemical physics* **1996,** *105* (5), 1902-1921.
6. Manual, D. U., Desmond2. 2. **2009**.
7. Martyna, G. J.; Klein, M. L.; Tuckerman, M., Nosé–Hoover chains: The canonical ensemble via continuous dynamics. *The Journal of chemical physics* **1992,** *97* (4), 2635-2643.
8. Martyna, G. J.; Tobias, D. J.; Klein, M. L., Constant pressure molecular dynamics algorithms. *The Journal of chemical physics* **1994,** *101* (5), 4177-4189.
